# Fast and tortuous: extreme deformations and fast movements of the cell nucleus are driven by microtubules in the model fungus *Podospora anserina*

**DOI:** 10.1128/mbio.01455-26

**Published:** 2026-07-31

**Authors:** Marie-caroline Viron, Zoé Kachaner, Pierre Grognet, Christophe Lalanne, Antoine Guichet, Sylvain Brun

**Affiliations:** 1 Université Paris Cité, CNRS-UMR 7592, Institut Jacques Monod (IJM) 555089, Paris, France; 2 Université Paris-Saclay, CEA, CNRS-UMR9198, Institute for Integrative Biology of the Cell (I2BC) 27048, Gif-sur-Yvette, France; 3 Université Paris Cité, CNRS-UMR8236, Laboratoire Interdisciplinaire des Energies de Demain (LIED) 555089, Paris, France; Instituto Carlos Chagas, Curitiba, Brazil

**Keywords:** nucleus, filamentous fungi, sexual reproduction, *Podospora anserina*, microtubules, cytoskeleton, actin, mechanotransduction

## Abstract

Nuclear movement, positioning, and the mechanical constraints applied to the nucleus play important roles in cellular identity, physiology, and gene regulation. This process, known as nuclear mechanotransduction, is an emerging field of research, and its deregulation can lead to severe human pathologies. In this study, we examine the behavior of male nuclei during sexual reproduction in the model fungus *Podospora anserina*. Once inside the trichogyne (the hyphal extension of the ascogonium, the female gamete), male nuclei do not undergo karyogamy with the female nuclei present in the trichogyne but migrate from the trichogyne tip, where plasmogamy occurred, to the ascogonium at the center of the protoperithecium. Our study highlights the contrasting behaviors of the two nuclear types: female nuclei remain immobile and spherical, while male nuclei move rapidly and exhibit striking deformations and stretching. Trichogynes are branched and compartmentalized, consisting of articles separated by septa that communicate through the septal pores. We show that during their migration across these compartments, male nuclei undergo the most extreme movements, deformations, and speeds reported to date for nuclei in motion. We also investigated the role of the actin and microtubule (MT) cytoskeletons in male nuclei behavior. We demonstrate that while actin depolymerization does not impair male nucleus migration, MT disruption causes a complete arrest of both nuclear movement and stretching, indicating a critical role for MTs. Finally, we reveal that the MT network in trichogynes is highly polarized, offering new insights into how male nuclei navigate within a complex branched network.

**IMPORTANCE:**

Here, using live-cell fluorescence imaging, we report the remarkable and complex behavior of male nuclei during sexual reproduction in the model fungus *Podospora anserina*. In particular, during their migration in female trichogynes, male nuclei undergo the most extreme movements, deformations, and speeds reported to date for nuclei in motion while female nuclei remain immobile. The highest speed reached by male nuclei is 135 µm/min and the most extreme stretching while passing by obstacles is 71 µm, which represents 12 times its size in a resting state. Furthermore, we show that microtubules (MT) are responsible for male nucleus movements and deformations and that the MT network is dense and highly polarized all along trichogynes. This latter discovery gives new insights into how nuclei eventually reach their destination, the protoperithecium, and how they can change direction and orientate in complex, ramified, and compartmentalized cells such as trichogynes.

## INTRODUCTION

The study of the cell nucleus has always been central to biology. Research into cell signaling and gene expression regulation has been pivotal in understanding how cells function, how they perceive their environment, how tissues organize, how organisms develop, and how diseases arise from impaired signaling or disrupted gene expression. In comparison, the understanding of mechanotransduction—that is, how physical forces and constraints applied to the nucleus can alter its organization and change gene expression—has only recently emerged as a new and growing field of research (1–5). The idea that signals transmitted to the nucleus could be mechanical in nature was largely ignored for a long time. However, an increasing number of human diseases are now being recognized as resulting primarily from failures in mechanotransduction (6, 7). Similarly, defective nuclear positioning has been linked to severe disorders such as microcephaly and myopathies (6, 8). Furthermore, abnormalities in nuclear envelope (NE) stiffness are associated with serious pathologies such as progeria and cancer (7, 9). Interestingly, it has been reported that reducing microtubule (MT)-generated forces on the NE can rescue defects associated with laminopathies (10). Nuclear deformation also plays a critical role in cancer cell dissemination. Because the nucleus is the largest and stiffest organelle in the cell, it becomes a limiting factor during cancer cell migration through tissue (11). The nuclei of metastatic cells undergo extreme deformation as they squeeze through tight spaces when penetrating or exiting the blood vessels for instance. Recent studies have shown that repeated nuclear deformation affects NE stiffness, chromatin organization, and gene expression, contributing to increased tumor aggressiveness (12). Mechanotransduction and nuclear movement have been studied in a variety of model organisms, ranging from yeast and Drosophila to humans. These studies have shown that nuclei interact with various cytoskeletal elements and that their movements are dependent on them. Unsurprisingly, MTs, actin, and their associated motor proteins are involved in both nuclear movement and mechanotransduction (5, 13–15). Lamins which play a crucial role in nuclei stiffness are also involved in these processes (7); however, lamins have not been identified in fungi, raising questions about how fungal nuclei resist mechanical constraints.

**Fig 1:**
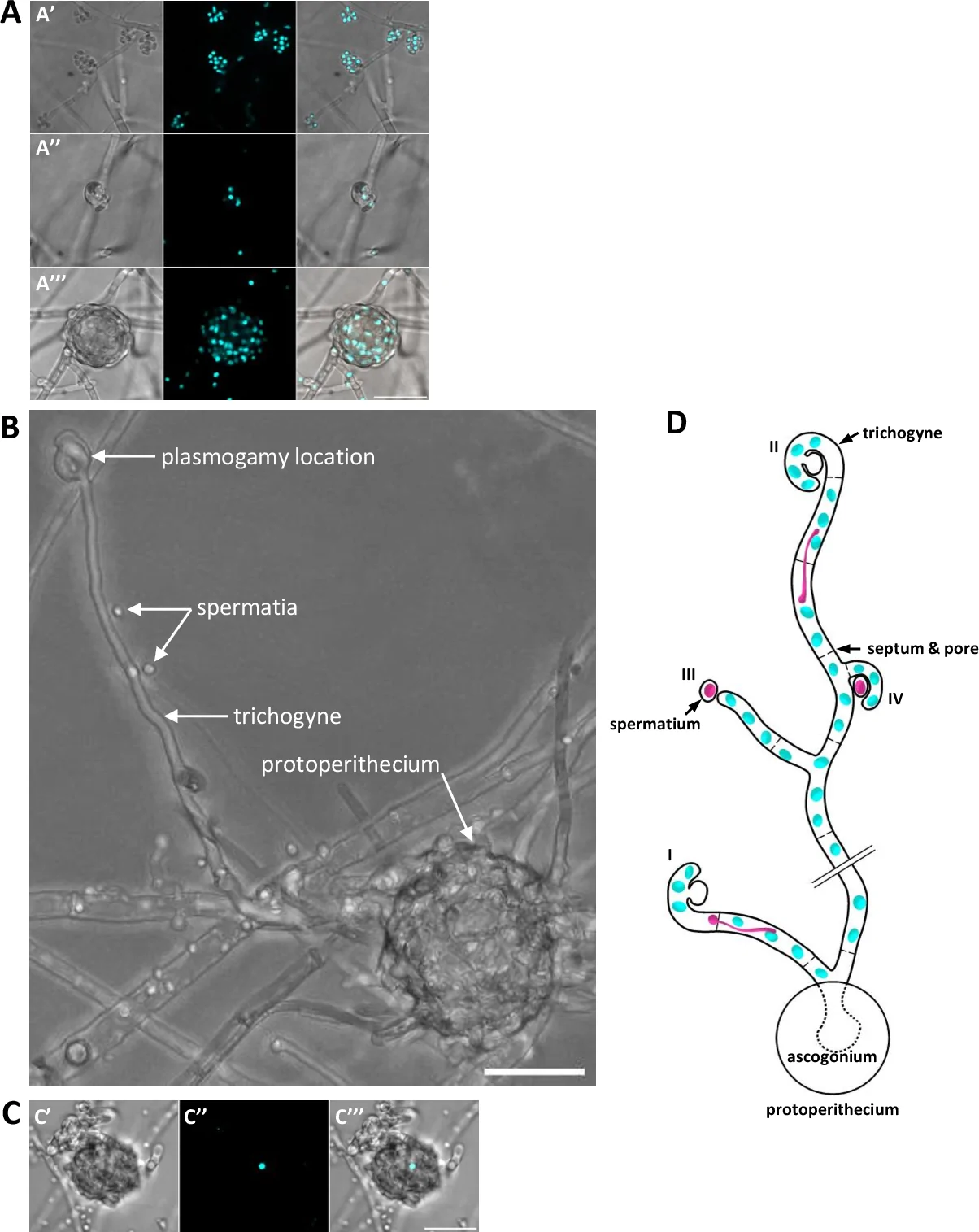
Reproductive organs engaged in sexual reproduction in *P. anserina*. (**A**) *H1-GFP* strain. (**A’**) conidiophores bearing spermatia; (**A’’**) ascogonium; (**A’’**’) protoperithecium. Images are shown from left to right: transmitted light, GFP fluorescence, and merged channels. (**B**) Z-projection image of a protoperithecium undergoing fertilization. The spermatium, bound by the trichogyne, is concealed by the trichogyne itself and is not visible in the image. (**C**) Wild-type protoperithecium fertilized by an H1-GFP-labeled male nucleus. Images from left to right: transmitted light (**C’**), GFP fluorescence (**C’’**), and merged channels (**C’’’**). Scale bars: 20 µm. (**D**) Schematic representation of the fertilization process in *P. anserina* using a cross between male *H1-mCherry* and female *H1-GFP* strains. The ascogonium, the “female” cell, lies at the core of the protoperithecium and produces trichogynes—sex-specific hyphae guided by pheromones released by “male” spermatia of the opposite mating type (19, 21). Spermatia contain a single nucleus. Trichogynes consist of a series of compartments (articles) separated by septa with septal pores that allow cytoplasmic continuity. Female nuclei (cyan) are distributed along the trichogyne and may undergo mitosis during trichogyne growth. We distinguish four modes of spermatium binding by trichogynes: Type I, binding by a newly growing trichogyne emerging from the protoperithecium; Type II, binding by the main apex of a trichogyne already extended on the medium at the time of spermatium inoculation; Type III, binding by a trichogyne branch specifically attracted to the spermatium; Type IV, binding by a small hook-like protrusion emerging from the trichogyne. After plasmogamy, male and female nuclei do not immediately fuse (karyogamy). Instead, male nuclei (magenta), contributed by spermatia attached at various points, migrate up the trichogyne toward the ascogonium. During this migration, male nuclei undergo significant deformation and elongation as they navigate through septal pores and bypass female nuclei.

In filamentous fungi, nuclear movement was first studied in vegetative hyphae (13, 16) and more recently during sexual reproduction in the model fungus *Neurospora crassa* (17). Here, we investigate this process in the model fungus *Podospora anserina*, a well-established organism for genetic and cell biology analysis (18). Its short life cycle (7 days), the production of small, easily imaged fruiting bodies and the ease of generating transgenic lines facilitated our cell biology and genetics approaches. In *P. anserina,* sexual reproduction between individuals of opposite mating types (heterothallic cross) is initiated when “female” trichogynes are attracted to pheromones secreted by “male” spores (spermatia in *P. anserina* and conidia in *N. crassa*) as in *N. crassa* (19). Eventually, the two structures fuse which represent the fertilization/plasmogamy step *per se*. Trichogynes are specialized sexual hyphae emitted by ascogonia, the female structures embedded in developing protoperithecia (fruiting bodies) (Fig. 1). These trichogynes, which can extend over hundreds of micrometers, are composed of successive compartments (called articles) separated by septa and connected via pores similarly as vegetative hyphae. Trichogynes contain female nuclei and, importantly after plasmogamy, the male nuclei that enter the trichogynes do not fuse with female nuclei but migrate toward the ascogonia (20).

Here, we provide a detailed description of the fertilization process, as well as of male nucleus movements and deformations during migration in *P. anserina*. During their migration, male nuclei exhibit an “inchworm-like” movement—repeatedly contracting and stretching as they bypass female nuclei or pass-through septal pores (Fig. 1D). Our study shows that the overall process is conserved compared to *N. crassa*, but we observed notable differences, including the frequent entry of multiple nuclei into protoperithecia and more dramatic speeds and deformations of male nuclei in *P. anserina*. We also investigated the actin and MT networks in trichogynes and their respective roles in male nucleus migration. Finally, we demonstrate that the MT network in trichogynes is highly polarized and that the remarkable movements, orientation, and deformations of male nuclei are dependent on MTs.

## RESULTS

### The male and female structures involved in sexual reproduction

To gain insights into the complete fertilization process in *Podospora anserina*, we performed live imaging of fertilization at low magnification (25× or 40× objectives), collecting data throughout all stages—from trichogyne growth to the eventual arrival of male nuclei in the protoperithecia. The fungal strains used in our experiments were genetically homogeneous, with all nuclei within an individual fungal thallus being genetically identical (homokaryotic). These homokaryotic *P. anserina* strains are hermaphroditic (Fig. 1), differentiating both “female” ascogonia (Fig. 1A'') and “male” spermatia (Fig. 1A'). Spermatia are mitospores similar to conidia; they are produced by conidiophores but, unlike typical conidia, cannot germinate. Instead, they function exclusively as “male” donor cells during fertilization (21). Ascogonia initially appear as twisted hyphal branches a few micrometers in length and are rapidly surrounded by adjacent vegetative hyphae (maternal tissue), forming protoperithecia (Fig. 1A''' and C) (22). While the protoperithecia gradually expand to reach diameters of several tens of micrometers, the ascogonia embedded within them produce trichogynes. In the absence of fertilization by spermatia of the opposite mating type, these trichogynes continue to grow and branch (Fig. 1B and D). Consequently, a fertile female network interwoven with vegetative hyphae was present in the 5- to 7-day-old “female” thalli used in our study. This fertile network, composed of trichogynes emerging from ascogonia, can only be fertilized by spermatia of the opposite mating type. *P. anserina* homokaryotic strains possess a single mating type—either *mat−* (*MAT-1-1*) or *mat+* (*MAT-1-2*)—and sexual reproduction occurs exclusively between strains of opposite mating types (heterothallism). In our experiments, we used *mat+* spermatia to fertilize *mat−* female mycelium, and, conversely, *mat−* spermatia to fertilize *mat+* mycelium. We did not observe any difference between the two reciprocal crosses. Therefore, for the sake of simplicity, the mating types of the strains used are not specified further in the manuscript.

### Trichogyne chemotropic growth and spermatium binding

**Fig 2:**
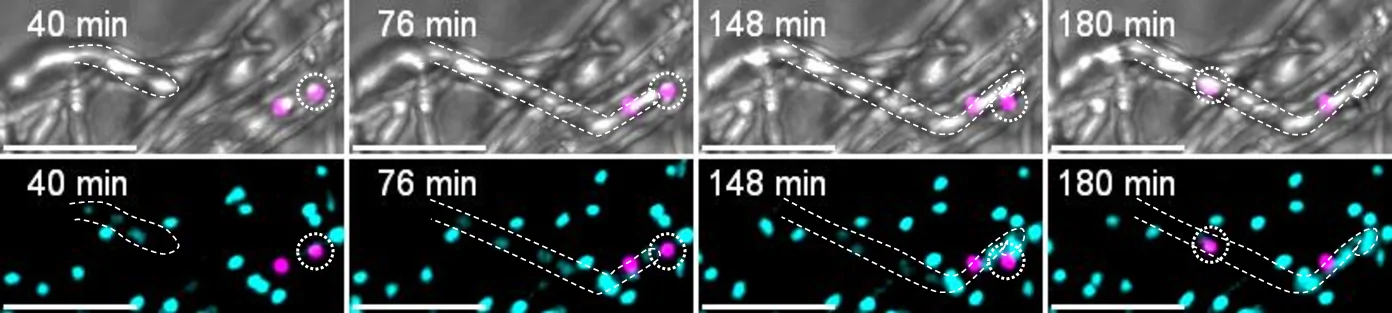
Trichogyne chemotropic growth followed by plasmogamy and male nucleus migration. Cross: male *H1-mCherry* × female *H1-GFP*. Z-projection images from time-lapse Movie 1 at https://doi.org/10.6084/m9.figshare.32685855 acquired using a spinning disk microscope (1 frame every 4 min). In the first row, transmitted light and mCherry channels are merged to visualize H1-mCherry-labeled male spermatia and nuclei (magenta). In the second row, GFP and mCherry channels are merged to simultaneously visualize H1-GFP-labeled female nuclei (cyan) and H1-mCherry-labeled male nuclei (magenta). The growing trichogyne is outlined with a dashed line, and the spermatium attracting the trichogyne is circled with a dashed line. At *t* = 76 min, the trichogyne establishes contact with the H1-mCherry-labeled spermatium. At *t* = 148 min, the H1-mCherry-labeled male nucleus has just entered the trichogyne. At *t* = 180 min, the H1-mCherry-labeled male nucleus (dashed circle) is migrating along the trichogyne toward the protoperithecium, located just outside the left edge of the field of view. Scale bar: 20 µm.

In our experiments, we tracked male nuclei inside spermatia or during their migration, as well as female nuclei inside trichogynes, by tagging nuclear chromatin with GFP-tagged (H1-GFP) and mCherry-tagged (H1-mCherry) histone H1 (see Materials and Methods, Table 1). When spermatia were inoculated onto the “female” thallus (also referred to as the female partner or mycelium) of the opposite mating type, we observed trichogynes (or their branches) growing toward the spermatia in an undulating manner reminiscent of chemoattraction, at a speed of 0.6 ± 0.3 µm/min (Fig. 1B and D and 2; Movie 1 at https://doi.org/10.6084/m9.figshare.32685855 [all the movies are downloadable from Figshare: https://figshare.com/; DOI: 10.6084/m9.figshare.32685855] and Table S1). Movie 1 illustrates the growth of a trichogyne eventually binding a spermatium (circled in Fig. 2), culminating in the migration of the male nucleus up the trichogyne (Fig. 2). As commonly observed, the trichogyne pushed and displaced the spermatium by a few micrometers after binding (*t* = 76 min). Seventy-two minutes later (*t* = 148 min), we observed the entry of the male nucleus expressing H1-mCherry into the trichogyne, followed by its migration toward the protoperithecium. As shown in Movie 1, the female nuclei residing within the trichogyne could undergo mitosis during trichogyne growth. In contrast, we never observed mitosis of female nuclei in trichogynes that had ceased growing due to spermatium binding.

**TABLE 1:** Strains genotypes. hygR: hygromycin resistant; phleoR: phleomycin resistance; nouR: nourseotricin resistance

Strain	Plasmid/genotype	Marker	Reference
Wild type	*big "S"*	None	(23)
*PaPks1^193^*	*PaPks1^193^*	None	(24)
*GFP^cyto^*	*pBC-hygR-AS4-GFP*	hygR	(25)
*H1-mCHerry*	*pBC-hygR-H1-mCherry*	hygR	This study
*H1-GFP*	*pBC-hygR-H1-GFP*	hygR	
*H1-mCherry GFP^cyto^*	*pBC-hygR-H1-mCherry pBC-hygR-AS4-GFP*	hygR	
β*-Tubulin-GFP*	*pBC-phleoR-AS4-*β*-Tubulin-GFP*	phleoR	
β*-Tubulin-mCherry*	*pBC-phleoR-AS4-*β*-Tubulin-mCherry*	phleoR	
*H1-mCherry* β*-Tubulin-GFP*	*pBC-hygR-H1-mCherry, pBC-phleoR-AS4-*β*-Tubulin-GFP*	hygR phleoR	
*LifeAct-GFP*	*pBC-hygR-AS4-LifeAct-GFP*	hygR	
*H1-mCHerry LifeAct-GFP*	*pBC-hygR-H1-mCherry, pBC-hygR-AS4-LifeAct-GFP*	hygR	
*PH-mCherry*	*pBC-nouR-AS4-PH-mCherry*	nouR	
*H1-GFP PH-mCherry*	*pBC-hygR-H1-GFP, pBC-nouR-AS4-PH-mCherry*	hygR nouR	

We observed that trichogynes could bind to spermatia in different ways. Spermatia could be bound by (i) the apex of newly emerging trichogynes projecting directly from protoperithecia (Type I), (ii) the principal apex of pre-existing trichogynes on the thallus at the time of inoculation (Type II), (iii) the apex of branches that developed on trichogynes and grew toward spermatia (Type III), or (iv) small hyphal hooks emerging from trichogynes and binding spermatia already in contact with the trichogyne (Type IV) (Fig. 1D). Importantly, we never observed spermatium/trichogyne binding involving trichogynes growing from young, bare ascogonia. Fertilization was only observed between spermatia and trichogynes emanating from properly formed protoperithecia. We further determined that the smallest protoperithecia fertilized by male nuclei had a diameter of 21 µm.

### Plasmogamy

**Fig 3:**
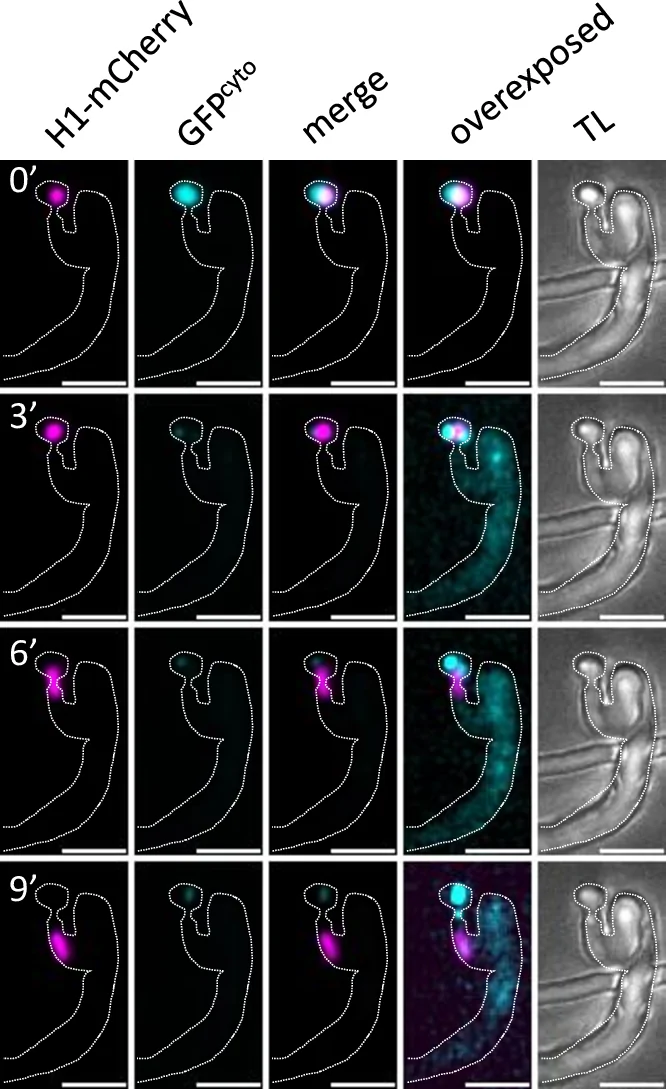
Plasmogamy followed by male nucleus entry into the trichogyne. Cross: male *H1-mCherry GFP^cyto^* × female wild type. Z-projection images from time-lapse Movie 2 at https://doi.org/10.6084/m9.figshare.32685855 acquired using a spinning disk microscope (1 frame every 3 min). The trichogyne and the spermatium fused together are outlined with a dashed line. At *t* = 0 min, both GFP^cyto^ and H1-mCherry signals are visible in the spermatium. At *t* = 3 min, the GFP^cyto^ signal almost completely disappears from the spermatium while simultaneously appearing in the trichogyne (visible in the overexposed panel), indicating that plasmogamy has occurred. At *t* = 6 min, the H1-mCherry-labeled male nucleus begins to enter the trichogyne. TL: transmitted light. Scale bar: 10 µm.

The initial step of plasmogamy described above—namely, the binding of trichogynes to spermatia—occurred serendipitously on female thalli following the inoculation of spermatia of the opposite mating type. To image the entire fertilization process, live—from trichogyne growth to the arrival of male nuclei in protoperithecia—while addressing its inherent unpredictability, we conducted live-recording sessions at low magnification. These recordings captured both protoperithecia and spermatia within the field of view over extended periods (10 to 15 h), using spermatia expressing H1-mCherry or H1-GFP. This protocol enabled us to document the complete fertilization process as previously described and to collect valuable data on its various stages. Notably, we determined that the average time between trichogyne–spermatium binding and the entry of the male nucleus into the trichogyne was 89 ± 33 min (Table S1). However, under these experimental conditions, we were unable to directly observe the cell fusion event that precedes male nuclei entry into the trichogyne. To address this limitation, we used *H1-mCherry GFP^cyto^* spermatia expressing H1-mCherry tagging the nucleus and a cytoplasmic GFP tagging the cytoplasm (GFP^cyto^) (25) (see Materials and Methods; Table 1). Following inoculation of these double-tagged spermatia, we tracked male nuclei before and during migration based on H1-mCherry fluorescence and we observed that the green fluorescent signal corresponding to the GFP^cyto^ was lost ~2 min 36 s (*n* = 5), as an average, before the onset of male nucleus migration (Fig. 3; Movie 2 at https://doi.org/10.6084/m9.figshare.32685855). As shown in Fig. 3, the loss of GFP^cyto^ fluorescence inside spermatia was due to the immediate dilution of spermatial cytoplasmic content into the trichogyne cytoplasm, marking the precise moment of cell fusion (especially visible in the overexposed merge panel of Fig. 3). This showed that male nuclei entry into trichogynes occurred relatively rapidly after cell fusion in comparison to the ~90 min pending time after initial trichogyne–spermatium binding.

### Entry of male nuclei into protoperithecia

Successful sexual reproduction relies on the eventual entry of the migrating male nucleus into the ascogonium in the core of the protoperithecium, or in other words, the “fertilization” of the protoperithecium which will develop into perithecium. As previously mentioned, trichogynes are often branched, allowing them to fuse with multiple spermatia, which may eventually lead to the entry of multiple male nuclei into a single protoperithecium (Fig. 1D). To quantify the “fertilization” rate of protoperithecia (entry of one male nucleus) and assess the occurrence of “polyfertilization” (entry of multiple nuclei), we conducted live imaging of the fertilization process (male *H1-mCherry* × female wild type [WT]) over a 15-h period. Recordings were performed at low magnification (25 × objective), beginning 4 h after spermatium inoculation. We observed that 70% of the recorded protoperithecia (16 out of 23) were “fertilized,” and notably, half of these (8 out of 16) were “fertilized” by more than one nucleus during the time-lapse (Fig. S1A; Movie S1 at https://doi.org/10.6084/m9.figshare.32685855). In Movie S1 (male *H1-mCHerry* × female *H1-GFP* cross), three male nuclei entered the same protoperithecium. In one case, we observed five nuclei entering a single protoperithecium (data not shown). This observation raised the question of whether each male nucleus entering a protoperithecium eventually engages in the subsequent steps of sexual reproduction, namely, karyogamy, meiosis, and ascospore production. Hence, could fertilization by spermatia with distinct genotypes lead to the formation of perithecia producing asci of different paternal origin, resulting in genetically mosaic ascospore contents? To tackle this question, we fertilized the wild-type *S* strain (which produces dark melanized ascospores) with a mixture of wild-type spermatia and spermatia from the *PaPks1^193^* mutant (which produces unpigmented, non-melanized ascospores) (24). We dissected 158 perithecia and found the following: 102 perithecia contained only asci with 4 melanized ascospores, indicating fertilization by wild-type spermatia. Fifty-six perithecia contained asci with two melanized and two non-melanized ascospores, consistent with fertilization by *PaPks1^193^* spermatia. No perithecia displayed a mosaic composition containing both types of asci (Fig. S1B). This result indicates that, despite frequent potential “polyfertilization” with multiple male nuclei entry, mature perithecia solely give rise to asci derived from a single paternal genotype.

### Difference between female and male nucleus behavior in trichogynes

**Fig 4:**
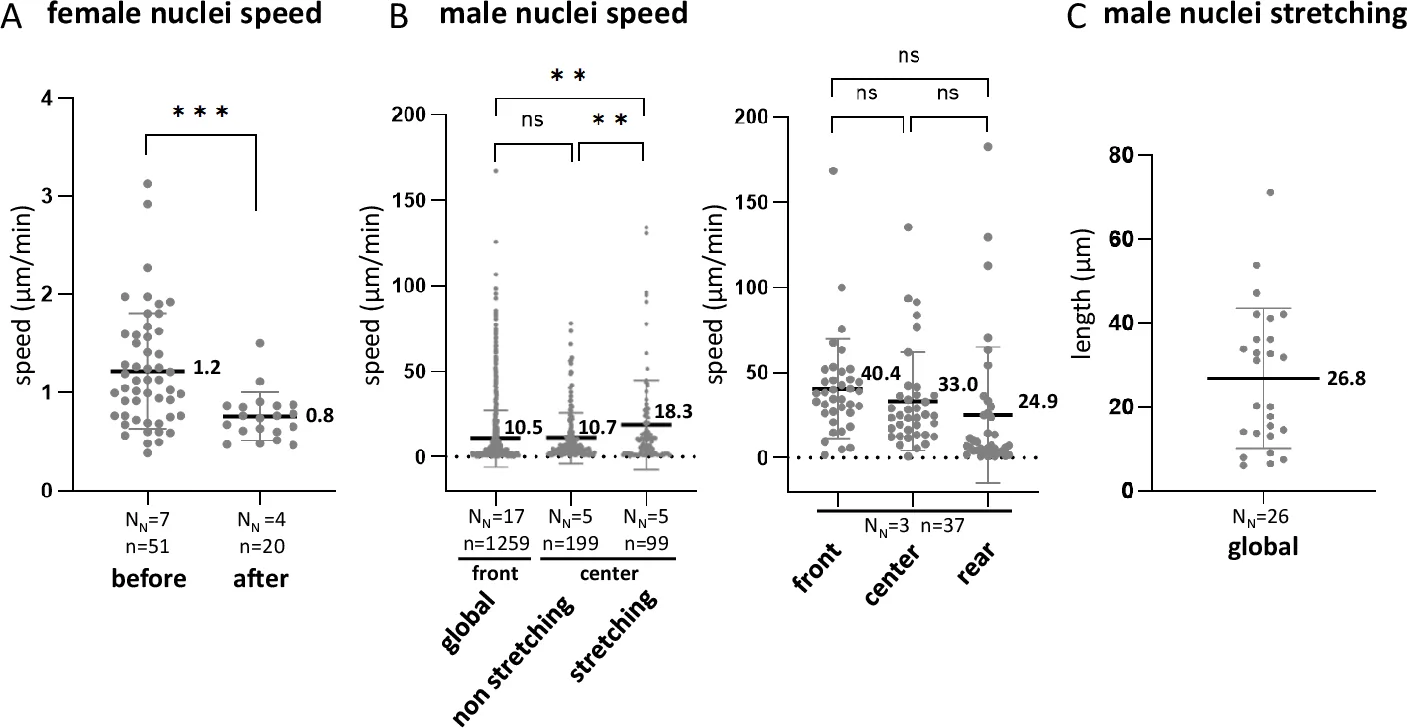
Speeds of female and male nuclei and male nuclei stretching during migration. (**A**) Cross: male *H1-mCherry* × female *H1-GFP*. Comparison of female nuclei speed before plasmogamy and after plasmogamy, during the migration of male nuclei; mean speeds are indicated on the graph. (**B**) Global: compilation of male nuclei front speed measurements from several crosses: male *H1-GFP* × female *H1-mCherry*, male *H1-mCherry* × female β*-Tubulin-GFP*, male *H1-GFP* × female *PH-mCherry*, and male *H1-mCherry* β*-Tubulin-GFP* × female wild type. Non-stretching and stretching: center speed measurements of male nuclei from *H1-mCherry* × female β*-Tubulin-GFP* crosses, divided into two categories—non-stretching: time points where the nuclei were in motion without stretching; stretching: time points showing active stretching. Front, center, and rear speed measurements are compiled from crosses involving male *H1-GFP* × female *PH-mCherry*, male *H1-mCherry* × female *LifeAct-GFP*, and male *H1-mCherry* × female β*-Tubulin-GFP* (see also Fig. S3). (**C**) Global: compilation of the maximum stretching length of male nuclei from crosses including *H1-mCherry* × female β*-Tubulin-GFP*, male *H1-GFP* × female *PH-mCherry,* and male *H1-mCherry* β*-Tubulin-GFP* × female wild type. For each nucleus, the greatest stretching length observed during the movie was recorded. *N*_N_: number of nuclei analyzed; *n*: number of individual speed measurements. Statistical analysis: Welch’s *t*-test. *P*-values: ns = not significant; *P < 0.05; **P < 0.01; ***P < 0.001; ****P < 0.0001.

**Fig 5:**
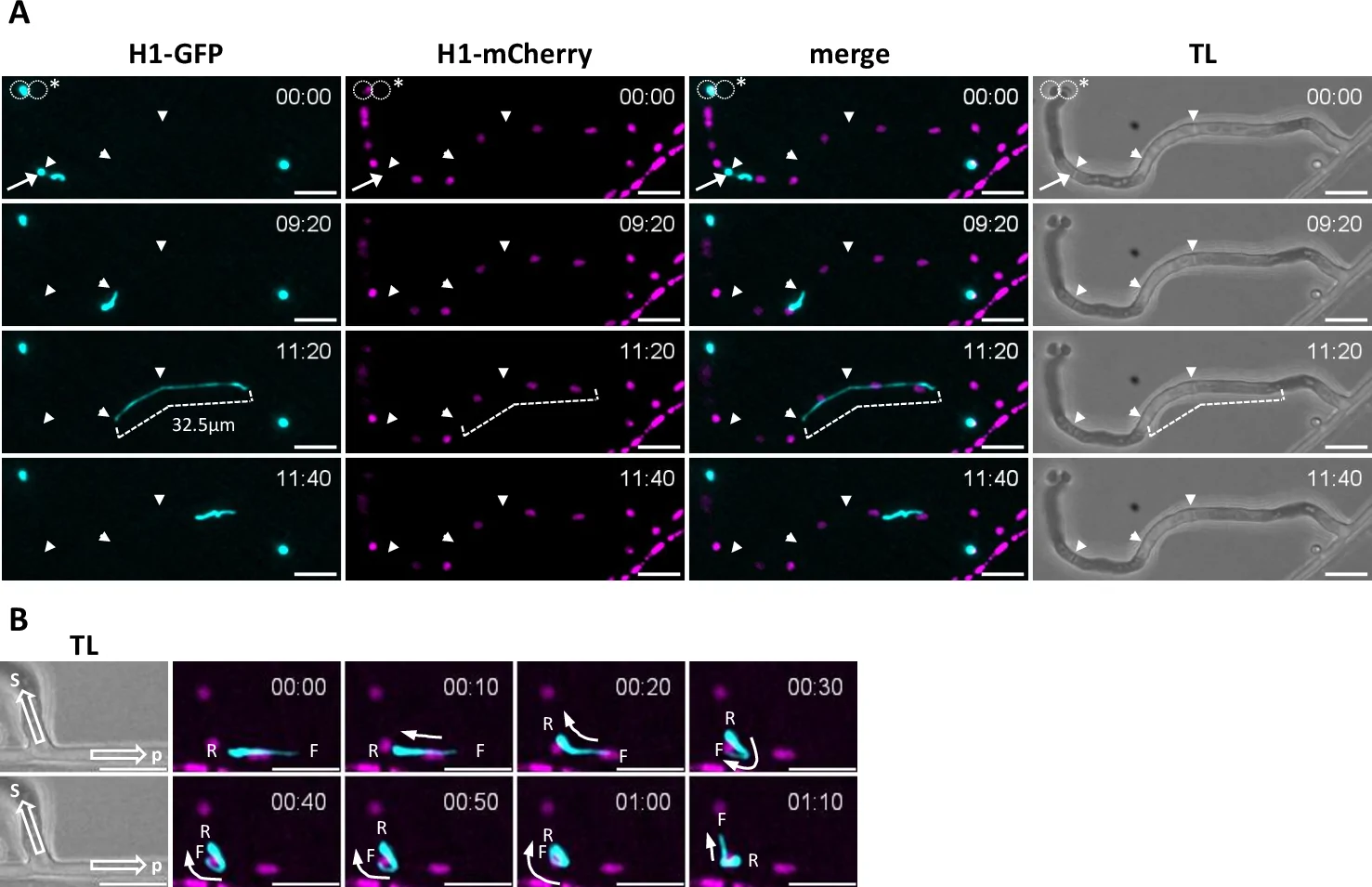
Male nucleus migration within trichogynes. Cross: male *H1-GFP* × female *H1-mCherry*. Z-projection images from time-lapse Movies 3 and 4 at https://doi.org/10.6084/m9.figshare.32685855 acquired using a spinning disk microscope (1 frame every 10 s). (**A**) Arrowheads indicate septa crossed by the H1-GFP-labeled male nucleus. In the *T* = 0 row, two spermatia are circled: the one on the left has not fused with the trichogyne and still contains its H1-GFP-labeled nucleus, while the one on the right marked with an asterisk has fused and released its nucleus, which is migrating up the trichogyne (arrow). At *t* = 0, the male nucleus is squeezed in the septal pore and appears split in two (but it is not). During migration, the H1-GFP-labeled nucleus exhibits back-and-forth movements and stretches while passing through the second and third septa during a single elongation phase; the maximum stretching observed is 32.5 µm (dashed line). H1-mCherry-labeled female nuclei remain stationary with only limited oscillatory movement. (**B**) A migrating H1-GFP-labeled male nucleus inside the trichogyne is shown. F: front of the nucleus; R: rear of the nucleus; arrow labeled "s" indicates the direction from which the nucleus originates (spermatium), and arrow labeled "p" indicates the direction toward the protoperithecium. Arrows show the direction of nuclear movement. From *t* = 10 s to *t* = 20 s, the nucleus reverses direction (simple backward movement); from *t* = 30 s to *t* = 1 min 10 s, it performs a U-turn, with the nucleus front changing direction. TL: transmitted light. Time format: min:s. Scale bar: 10 µm.

We crossed male and female strains expressing differentially tagged nuclei to enable the independent tracking of each nuclear type during fertilization. Notably, in both male *H1-mCherry* × female *H1-GFP* and male *H1-GFP* × female *H1-mCherry* crosses, we never observed any merging or overlap of the GFP and the mCherry fluorescence signal within the nuclei (Fig. 2). This allowed the clear and simultaneous observation of male and female nuclei within the same trichogynes. We first characterized the behavior of female nuclei in trichogynes, both before plasmogamy and after plasmogamy, during male nucleus migration. Prior to plasmogamy, female nuclei in trichogynes (i) were distributed along the entire length of the trichogynes, (ii) were round with an average diameter of 2.1 ± 0.2 µm, (iii) exhibited oscillatory movements, and (iv) underwent division exclusively during trichogyne growth (Table S1; Fig. 1D, 2, 4, and 5; Movies 1, 3, and 4 at https://doi.org/10.6084/m9.figshare.32685855). After spermatium binding, female nuclei within the terminal article of the trichogyne tended to accumulate near the tip (Fig. 2). Following plasmogamy, both male and female nuclei coexisted within the trichogyne: male nuclei migrated from the spermatia toward the protoperithecia, while female nuclei generally remained stationary. Interestingly, female nuclei in trichogynes undergoing male nucleus migration displayed a significant reduction in oscillatory speed compared to their behavior before plasmogamy (Fig. 4A). Unlike the predominantly round female nuclei, male nuclei were never round during migration. Instead, they displayed remarkable morphological plasticity, transitioning between relaxed and extremely elongated forms. In their relaxed state, male nuclei had a mean length of 6.2 ± 1.0 µm (Table S1), whereas the maximum observed length for a stretched male nucleus reached up to 71 µm (Fig. S2; Movie S2 at https://doi.org/10.6084/m9.figshare.32685855).

### Male nucleus migration

Male nucleus migration was a highly complex and discontinuous process, characterized by phases of acceleration, periods of stalling, back-and-forth movements, changes of direction at branch points, and notably, phases of extreme nuclear stretching. To accurately describe these movements, we assigned each male nucleus a front, center, and rear and analyzed their respective movements and speeds separately during migration (Fig. 4B and 5; Fig. S3). Our primary focus was on the front speed when describing male nucleus migration, but we also compared speeds at the center and rear to gain deeper insights into the nuclei’s complex dynamics. The average migration speed of male nuclei was highly variable, with a mean front speed of 10.5 ± 16.7 µm/min (Fig. 4B). Male nuclei frequently exhibited back-and-forth movements. Careful analysis revealed two types of direction changes: U-turns**,** when the front of the nucleus executed a 180° turn and remained the front after the reversal and “simple” reversals, when the rear of the nucleus became the new front (Fig. 5B; Movies 3 and 4 at https://doi.org/10.6084/m9.figshare.32685855). Note that we never observed male nuclei crossing septal pores in one direction and then crossing it back again, suggesting that reversals could be restrained within articles. In branched trichogynes, nuclei could enter one branch, change direction, and migrate into another (data not shown). Essentially, two kinds of movements were observed at both the front and rear ends of the nucleus as illustrated in Movie S3 at https://doi.org/10.6084/m9.figshare.32685855: traction movements reminiscent of pulling forces, and retraction movements reminiscent of an elastic reaction of the nucleus. The highest traction speed recorded was 125.4 µm/min, and it was for the front of the nucleus (male *H1-GFP* × female *PH-mCherry*, Fig. 4B; Movie S3). The highest retraction speed measured was for the rear of the nucleus: 248.9 µm/min (male *H1-mCherry* × female β*-Tubulin-GFP*, Movie 5 at https://doi.org/10.6084/m9.figshare.32685855).

To further characterize the overall movement of male nuclei, we compared the speeds of the front, center, and rear in three male nuclei during stretching phases (Fig. 4B; Fig. S3). Our first observation was that the mean speeds of these three regions did not differ statistically (Fig. 4B). Second, at every time point, the center speed consistently fell between the front and rear speeds (Fig. S3). Therefore, when the front was being pulled forward and the rear was retracting simultaneously, the speed measured at the center could be higher than at the front. Indeed, we recorded a maximum center speed of 135.2 µm/min**,** surpassing the previously noted maximum front speed of 125.4 µm/min (Fig. S3C). We also compared the center speed of male nuclei during stretching and non-stretching phases, excluding pausing intervals to evaluate whether stretching correlated with faster movement. When not stretching, nuclei moved at an average speed of 10.7 ± 14.8 µm/min, whereas during stretching, their speed increased significantly to 18.3 ± 26.2 µm/min (Fig. 4B and *t*-test, *P* = 0.008), indicating that nuclei move faster while stretched.

Male nuclei were observed contorting and elongating both when passing by bulky organelles, such as female nuclei and while crossing septal pores, with an average stretched length of ~27 ± 17 µm (; Fig. 4C and 5A; Fig. S2). The most extreme stretching measured reached 71 µm (male *H1-mCherry* × female β*-Tubulin-GFP*; Fig. S2; Movie S2 at https://doi.org/10.6084/m9.figshare.32685855), approximately 12 times their relaxed length and 35 times larger than regular vegetative or female nuclei. Among the different cell organelles, resident female nuclei and large vacuoles, along with septal pores, likely represent the principal physical obstacles to migrating male nuclei. Female nuclei, with a diameter of 2.1 ± 0.2 µm, occupy most of the trichogyne cross-sectional area, which averages 3.5 ± 0.6 µm (Table S1). Trichogynes are composed of compartments separated by septa (Fig. 1D). The diameter of septal pores in *P. anserina* vegetative hyphae is about 200 nm (data not shown). Assuming a similar size of septal pores in trichogynes, the relaxed male nuclei width of 0.8 ± 0.2 µm is roughly four times larger than the pores. Consistently, in nearly all recorded movies, male nuclei often paused before septa, slackening to relaxed form, then stretching again to pass through (Movies 3 to 5, 7, 8, 12, and S2 at https://doi.org/10.6084/m9.figshare.32685855). However, we also observed male nuclei crossing septal pores without pausing, sometimes crossing two septa consecutively while stretched (e.g., Fig. 5; Movie 3 at *t* = 11:20). This indicated that relaxation before pore passage was frequent but not obligatory. For instance, in Fig. 5A, the male nucleus crossed the second septum without stopping before (*t* = 11:20; Movie 3). Together, these data demonstrate that male nucleus migration is a complex process. Male nuclei migrate rapidly and undergo extreme deformation, likely necessary to bypass bulky obstacles such as female nuclei and, most importantly, to traverse septal pores.

### Actin depolymerization does not affect nucleus movements

**Fig 6:**
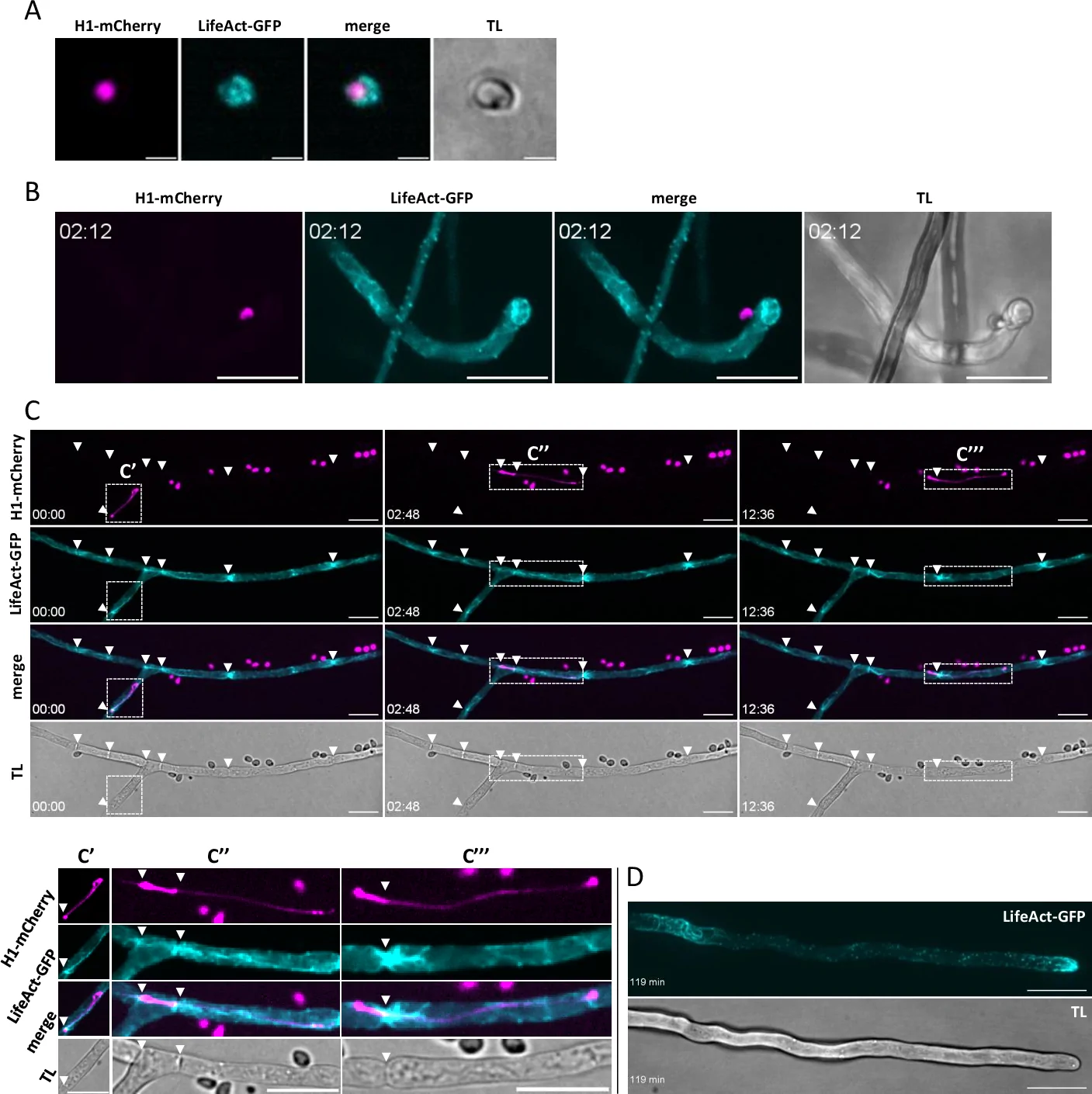
Actin cytoskeleton in spermatia, vegetative hyphae, and trichogynes. (**A**) Spermatium expressing H1-mCherry and LifeAct-GFP. Z-projection images showing actin organization. Scale bar: 2 µm. (**B**) Trichogyne prior to plasmogamy from a cross between male *H1-mCherry* and female *LifeAct-GFP*. Z-projection images from time-lapse Movie 6 at https://doi.org/10.6084/m9.figshare.32685855 acquired using a spinning disk microscope (1 frame every 12 s). The trichogyne has been attracted by the H1-mCherry-labeled spermatium in the field of view. Time format: min:s. Scale bar: 10 µm. (**C**) Trichogyne during male nucleus migration from a cross between male *H1-mCherry* and female *LifeAct-GFP*. Z-slices from time-lapse Movie 7 at https://doi.org/10.6084/m9.figshare.32685855 acquired using a spinning disk microscope (1 frame every 12 s). Movie 7 captures two H1-mCherry-labeled nuclei migrating sequentially within a LifeAct-GFP-labeled trichogyne. The first and second columns show the first migrating nucleus, and the third column shows the second. Panels C’, C’’, and C’’’ are high magnifications of the regions indicated by dashed rectangles. During migration, male nuclei show alternating phases of colocalization between H1-mCherry signal and LifeAct-GFP-labeled actin cables (**C’ and C’’**) and phases where colocalization is partial or absent (**C’’’**). Multiple spermatia containing H1-mCherry-labeled nuclei are visible in the field of view. TL: transmitted light. Scale bar: 10 µm. (**D**) Vegetative hyphae expressing LifeAct-GFP. Z-projection images from time-lapse Movie S4 at https://doi.org/10.6084/m9.figshare.32685855 acquired using a spinning disk microscope (1 frame per minute). Scale bar: 10 µm.

**Fig 7:**
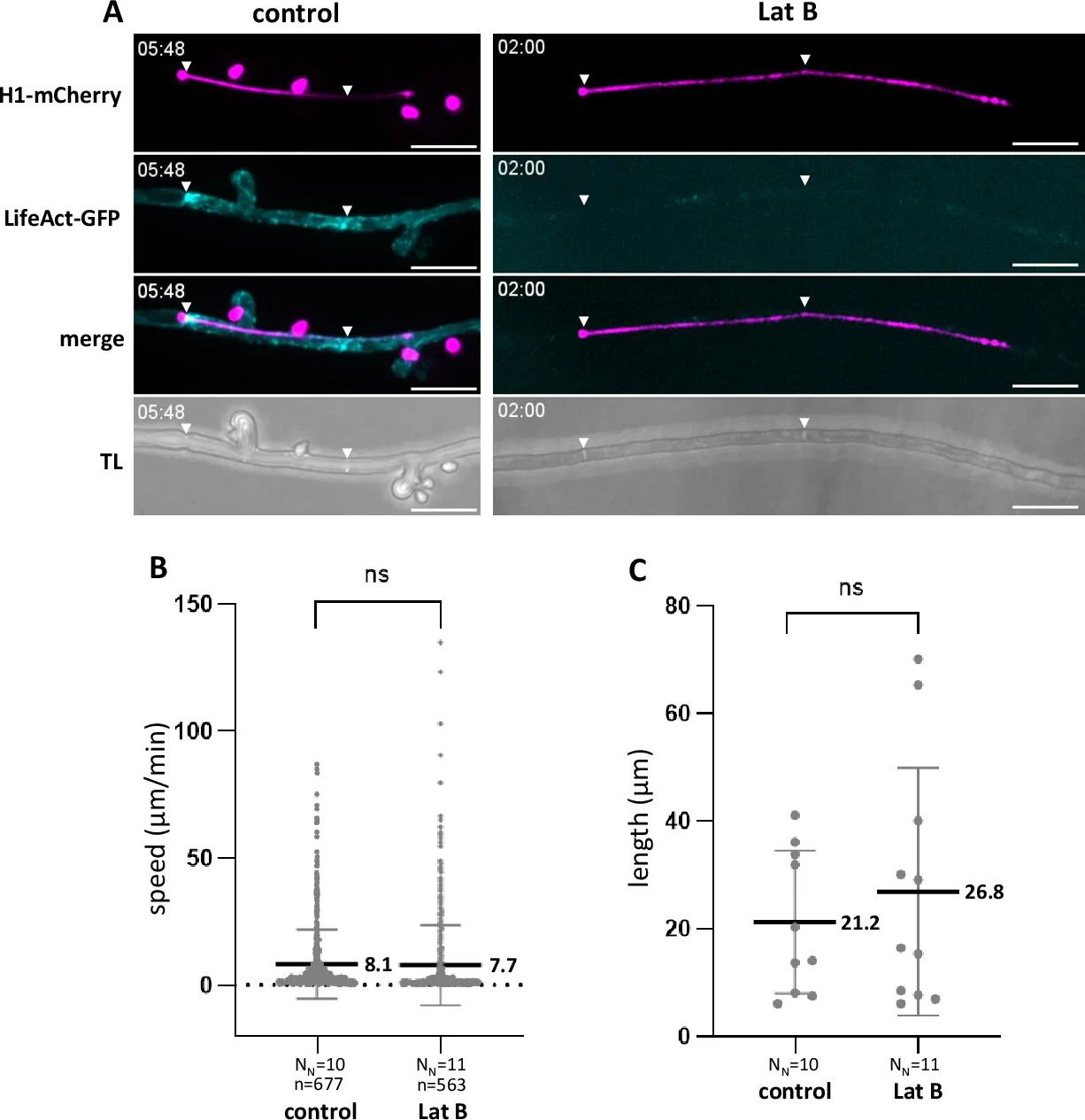
Actin depolymerization does not impair male nucleus movements and stretching. Cross: male *H1-mCherry* × female *LifeAct-GFP*, treated with 25 µM latrunculin B (Lat B) or untreated (control). (**A**) Z-projection images from time-lapse movies acquired on a spinning disk microscope. Control: Movie 8 at https://doi.org/10.6084/m9.figshare.32685855 (1 frame every 12 s); Lat B: Movie 9 at https://doi.org/10.6084/m9.figshare.32685855 (1 frame every 15 s). Despite Lat B-mediated depolymerization of actin filaments, male nucleus movement and stretching remain unaffected. In the control condition, four spermatia with H1-mCherry-labeled nuclei are visible within the field of view. Time format: min:s. Scale bar: 10 µm. (**B**) Male nuclei front speed. The front speed of male nuclei was measured at each time point throughout the time-lapse recordings. Mean speeds are indicated in the graph. No significant difference in speed was observed following actin depolymerization. *N*_*N*_: number of nuclei analyzed; *n*: number of individual front speed measurements. (**C**) Maximum male nuclei stretching. For each nucleus, the greatest stretching length observed during the movie was recorded. Mean maximum stretching lengths are shown in the graph. *N*_*N*_: number of nuclei analyzed. Control was treated with 0.3% DMSO. Welch’s *t*-test: ns = not significant.

The major cytoskeletal components responsible for nuclear movements in eukaryotes are actin filaments and microtubules (MTs). However, neither of these cytoskeletal elements has been studied in *P. anserina* to date. To investigate the actin network, we first constructed a LifeAct-GFP reporter that specifically binds filamentous actin (see Materials and Methods and Table 1) (26–28). We analyzed the actin network in LifeAct-GFP expressing vegetative hyphae and in *H1-mCherry LifeAct-GFP* spermatia. To study actin dynamics during fertilization, we crossed *H1-mCherry* males with *LifeAct-GFP* females and observed actin staining patterns in trichogynes before plasmogamy and during male nucleus migration after plasmogamy. Actin was present in spermatia (Fig. 6A) as well as in trichogynes both before and after plasmogamy during male nucleus migration (Fig. 6B and C; Movies 6 to 8 at https://doi.org/10.6084/m9.figshare.32685855). Prior to plasmogamy, the actin network in trichogynes (Fig. 6B; Movie 6) resembled that observed in vegetative hyphae (Fig. 6D; Movie S4 at https://doi.org/10.6084/m9.figshare.32685855), characterized by a high concentration of actin at the trichogyne tip, parsimonious filaments, and numerous actin patches. Filament formation was highly dynamic in both trichogynes and vegetative hyphae, consistent with previous observations in other fungi (29, 30). Remarkably, the actin network became much more prominent in trichogynes during male nucleus migration. It consisted of numerous patches but was dominated by longitudinal cables spanning the trichogyne compartments, with a particularly high accumulation at septa (Fig. 6C; Movies 7 and 8 at https://doi.org/10.6084/m9.figshare.32685855). Interestingly, we occasionally observed co-localization of H1-mCherry tagged, stretched male nuclei with LifeAct-GFP labeled actin cables, though this was not consistent during every nuclear stretching event (Fig. 6C). To test whether the actin network was essential for male nucleus movements, we treated samples with latrunculin B (Lat B), a drug that depolymerizes actin filaments (31), and monitored male nuclei dynamics in trichogynes. Four hours after inoculating *LifeAct-GFP* females with *H1-mCherry* spermatia (male *H1-mCherry* × female *LifeAct-GFP*), fertilized samples were mounted in 25 µM Lat B (Fig. 7; Movie 9 at https://doi.org/10.6084/m9.figshare.32685855), and both actin network integrity and male nucleus movements were assayed. Strikingly, complete actin depolymerization in Lat B-treated trichogynes (Fig. 7A) did not affect either male nuclei speed (Fig. 7B) or nuclear stretching (Fig. 7C; Movie 9). This indicates that actin filaments do not play a major role in male nucleus movements and deformation within trichogynes.

### MTs are polarized in trichogynes

**Fig 8:**
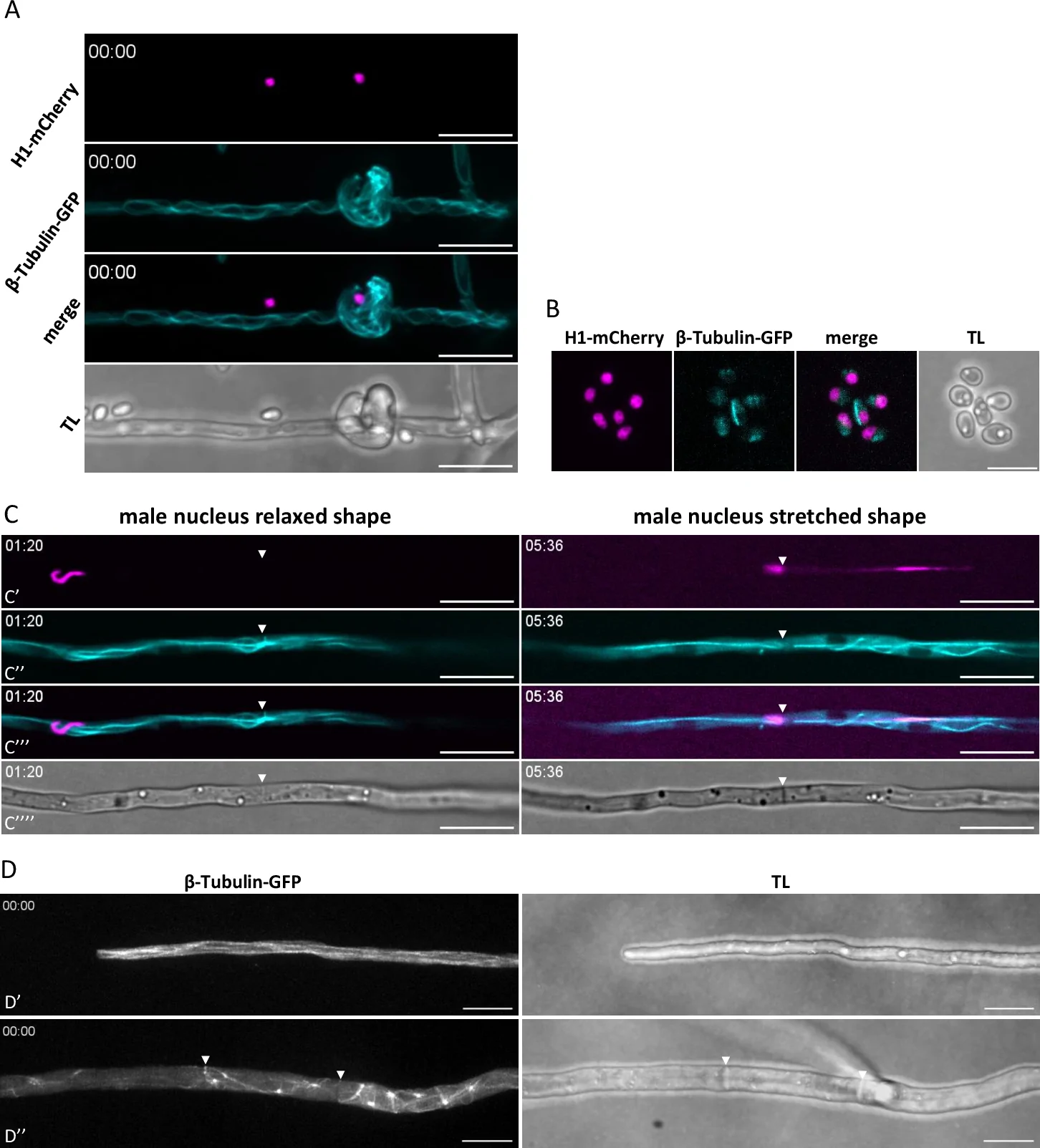
Microtubule network organization in *P. anserina*. (**A**) Microtubule (MT) organization in a trichogyne prior to plasmogamy, from a cross between male *H1-mCherry* × female β*-Tubulin-GFP*. Z-projection images from time-lapse Movie 10 at https://doi.org/10.6084/m9.figshare.32685855 (1 frame every 8 s). The spermatium on the right attracts the trichogyne, which coils around it. Time format: min:s. Scale bar: 10 µm. (**B**) MT labeling in spermatia expressing H1-mCherry and β-tubulin-GFP. Z-projection images. Scale bar: 5 µm. (**C**) MT organization in a trichogyne during male nucleus migration, from a cross between male *H1-mCherry* × female β*-Tubulin-GFP* (mounted in 1.1% DMSO). Z-slice images from time-lapse Movie 5 at https://doi.org/10.6084/m9.figshare.32685855 (1 frame every 8 s). Time format: min:s. (**C’**) H1-mCherry channel; (**C’’**) β-tubulin-GFP channel; (**C’’’**) merged channels; (**C’’’’**) transmitted light. The migrating H1-mCherry-labeled male nucleus moves from left to right. In the left panel, the nucleus is in a relaxed shape with no visible interaction between its front and MTs. In the right panel, the front of the nucleus is being pulled, the nucleus is stretched, and its front colocalizes with MTs. Arrowhead: septum. Scale bar: 10 µm. (**D**) MT organization in vegetative hyphae expressing β-tubulin-GFP. Z-projection images from time-lapse recordings: (**D’**) hyphal apex from Movie S5; (**D’’**) hyphal segment from Movie S6 at https://doi.org/10.6084/m9.figshare.32685855. Arrowheads indicate septa. Frame rate: 1 frame every 10 s. Time format: min:s. Scale bar: 10 µm.

MTs have never been studied in *P. anserina*. To investigate them, we constructed reporter strains in which the β-subunit (Pa_4_7630) of MT protofilaments is tagged with either GFP or mCherry (see Materials and Methods and Table 1). We analyzed the MT network in β*-Tubulin-GFP* vegetative hyphae and in *H1-mCherry* β*-Tubulin-GFP* spermatia. To study MT dynamics during fertilization, we crossed *H1-mCherry* as male with β*-Tubulin-GFP* as female and observed MT labeling in β*-Tubulin-GFP* trichogynes. The β-tβubulin-GFP labeled MT network in trichogynes was markedly different from that in vegetative hyphae. In vegetative hyphae, long longitudinal MT bundles were observed at the apex (Fig. 8D’; Movie S5 at https://doi.org/10.6084/m9.figshare.32685855), consistent with observations in other species (32). More proximally, the MT network appeared more scattered, displaying both longitudinal bundles and bundles originating from intensely GFP-labeled, motile foci (Fig. 8D’’; Movie S6 at https://doi.org/10.6084/m9.figshare.32685855). Notably, septa in vegetative hyphae showed little to no β-tubulin-GFP signal (see below).

**Fig 9:**
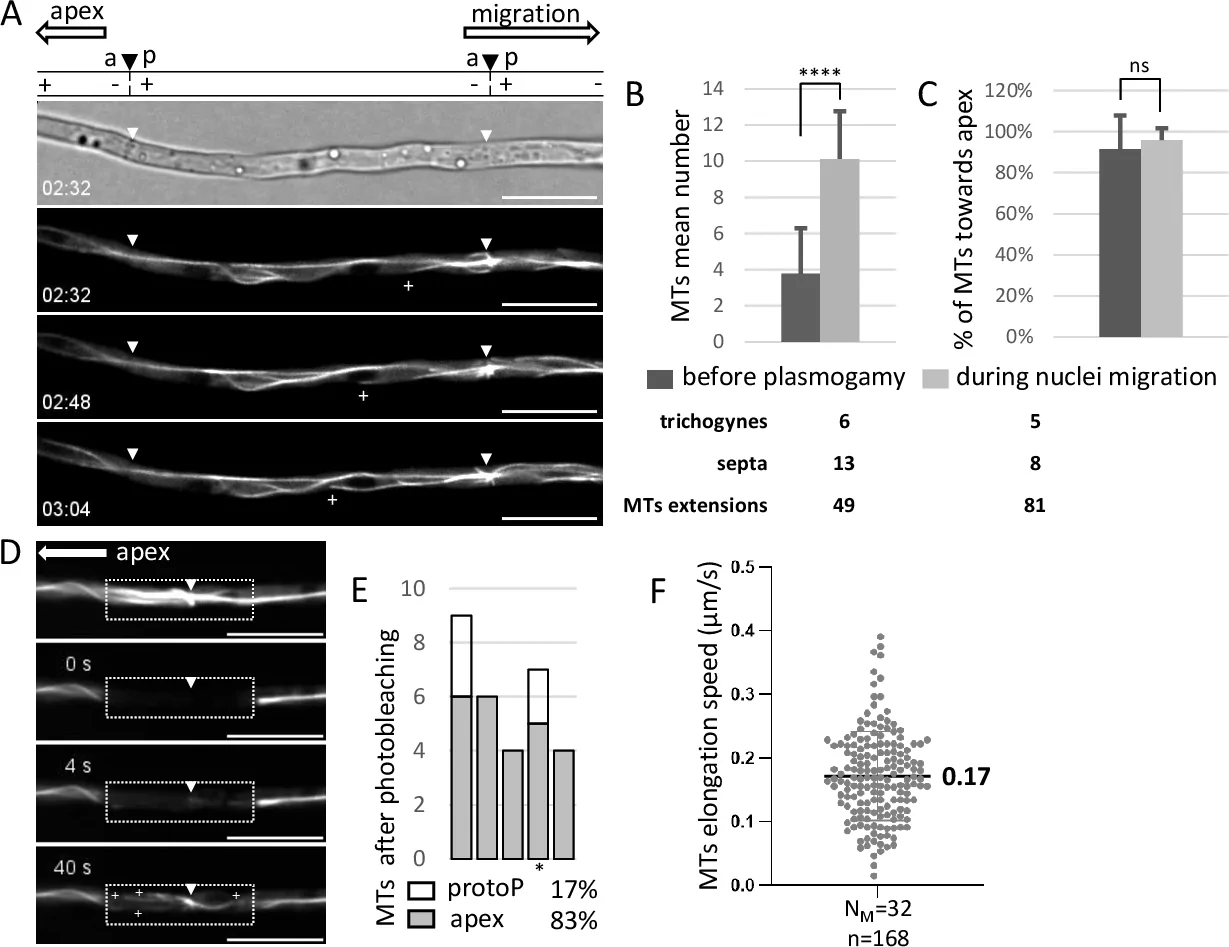
The microtubule network in trichogynes is polarized. Cross: male *H1-mCherry* × female β*-Tubulin-GFP* (mounted in 1.1% DMSO). (**A**) Z-slice images from time-lapse Movie 11 at https://doi.org/10.6084/m9.figshare.32685855 (1 frame every 8 s). Arrowheads indicate septa. At the top of panel A, the orientation of the trichogyne and the direction of male nucleus migration are shown. The schematic indicates septal orientation: “a” for apex side, “p” for protoperithecium side; “+” and “–” represent the main MT polarity within the trichogyne compartments as inferred from panels B and C. The first row shows transmitted light images; subsequent rows show β-tubulin-GFP labeling. Several MT bundle elongations are visible across the time points, with the (+) end of the most prominent one indicated by a “+.” Time format: min:s. Scale bar: 10 µm. (**B**) Quantification of the mean number of elongating MTs on both sides of septa before plasmogamy and during male nucleus migration (after plasmogamy). Welch’s *t*-test: ns = not significant; ****P < 0.0001. (**C**) Mean percentage of elongating MTs on the apical side of septa and directed toward the apex vs those directed toward the center of the thallus. No significant difference was found between pre- and post-fertilization percentages (*χ*² (1) = 2.23, *P* = 0.135). The number of trichogynes, septa, and elongating MTs analyzed before and after plasmogamy is indicated below the graphs. Counts were performed on a single z-slice over a 10-min recording window. The majority of MTs in trichogynes grow toward the apex, establishing a polarized network as illustrated in the schematic. (**D**) FRAP experiment performed on β*-Tubulin–GFP* trichogynes prior to *H1-mCherry* male nucleus migration (Z-slice images extracted from time-lapse Movie S7 at https://doi.org/10.6084/m9.figshare.32685855; one frame was acquired every 5 s before photobleaching and 4 s following photobleaching). The orientation of the trichogyne is indicated. The arrowhead marks the septum. The dashed rectangle outlines the photobleached region. (+) MT bundles plus-ends. Scale bar: 10 µm. (**E**) Each bar in the graph represents the number of MTs bundles growing within the photobleached region during the 4-min time-lapse recording (precisely on the right side of the septum, as described in Materials and Methods), counted in five different trichogynes. The numbers of MTs growing toward the apex and toward the protoperithecium (*n* = 30), as well as their overall percentages, are indicated. *, counting corresponding to Movie S7 at https://doi.org/10.6084/m9.figshare.32685855. (**F**) Speed of MT bundle elongation (µm/s); *N*_*M*_: number of MT bundle elongation events analyzed; *n*: number of individual speed measurements.

In contrast, MTs in trichogynes predominantly consisted of thick, long bundles appearing to traverse the length of the trichogyne (Fig. 8A, C and 9A; Movies 5 and 10 to 12 at https://doi.org/10.6084/m9.figshare.32685855). In fungi, spindle pole bodies (SPBs) as well as septa function as important microtubule-organizing centers (MTOCs) (32–34). As noted above, β-tubulin-GFP labeling at vegetative hyphal septa was weak (Fig. 8D’’). Accordingly, we rarely observed MT bundles growing from septa in vegetative hyphae: among 23 septa analyzed over 10-min recordings, only 1 MT bundle elongation was detected (data not shown). In sharp contrast, β*-Tubulin-GFP* trichogynes exhibited intense labeling at septa along with numerous MT bundle extensions primarily oriented toward the apex (Fig. 9A; Movie 11). Although tools for specifically tagging MT minus- or plus-ends are not available in *P. anserina*, these MT bundle extensions were clearly visible, allowing us to quantify them and determine their growth direction and speed (0.17 ± 0.07 µm/s) (Fig. 9F). We counted the number of MT bundle extensions at septa (within a single focal plane over 10 min) to calculate the percentage growing toward the apex vs toward the protoperithecium, thereby assessing MT polarity in trichogynes (Fig. 9B and C). Because MTs can cross septal pores, the extensions observed at septa may include both MTs polymerizing at septa and those crossing the pores. First, we observed that the average number of MT bundle extensions per septum was significantly higher during male nucleus migration (10.1 ± 2.6) compared to before plasmogamy (3.8 ± 2.5). More importantly, 92% ± 16% of these MT bundles grew toward the trichogyne apex before plasmogamy, and 96% ± 6% during male nucleus migration. This polarized growth of MTs was further investigated using fluorescence recovery after photobleaching (FRAP). Because septa represent major sites of MT nucleation in trichogynes, regions encompassing septa were selected for photobleaching. FRAP experiments were performed on β*-Tubulin–GFP* trichogynes prior to male nucleus migration (Fig. 9D; Movie S7 at https://doi.org/10.6084/m9.figshare.32685855). In all FRAP recordings, diffuse β-tubulin–GFP fluorescence was observed in the first frame acquired after photobleaching (4 s), indicating the nearly instantaneous diffusion of cytoplasmic fluorescent β-tubulin–GFP from both sides of the bleached region. This was rapidly followed by, or concomitant to, labeling of the septum and the appearance of the first MT bundle extremities marked by fluorescent β-tubulin–GFP within the photobleached area. We also could clearly see MTs passing septal pores. Importantly, in the five FRAP movies analyzed, the vast majority of MT bundles emerging within the photobleached region appeared to grow in the direction of the apex. However, because these bundles rapidly became numerous, counting events in the photobleached area located on the proximal side of the septum (right side) proved easier and more reliable (Fig. 9E). Using this approach, we determined that 83% (25/30) of the MT bundles appearing in this region actually grew toward the apex. Taken together, these data demonstrate that the MT network in trichogynes is highly dynamic and polarized both before and during male nucleus migration. Indeed, the vast majority of MT plus-ends extend toward the apex in the opposite direction of male nucleus migration.

### Male nucleus movements depend on MTs

**Fig 10:**
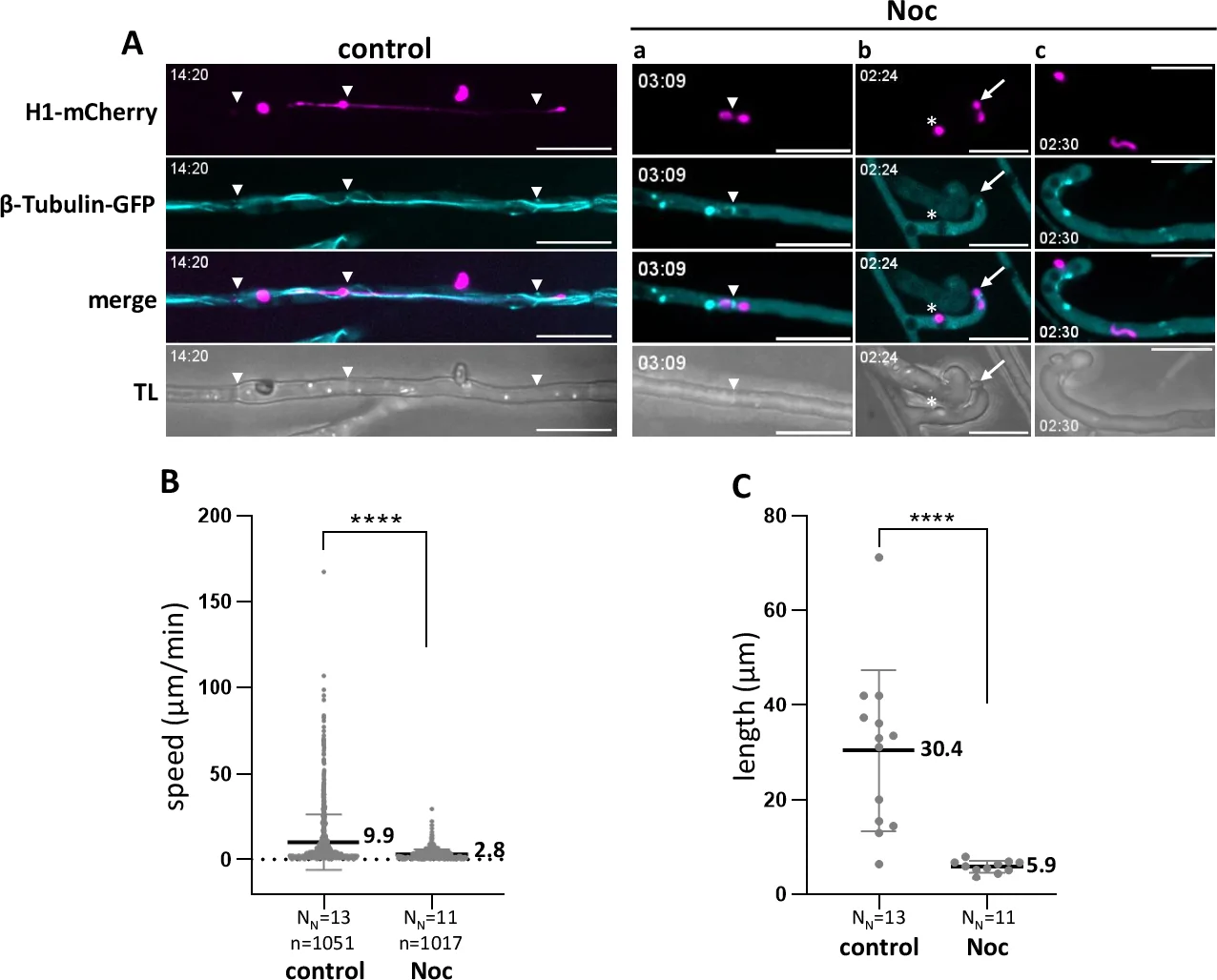
Male nucleus migration in trichogynes depends on microtubules. (**A**) Z-projection images from time-lapse movies acquired on a spinning disk microscope. Comparison between untreated controls (male *H1-mCherry* × female β*-Tubulin-GFP*, mounted in 1.1% DMSO; Movie 12 at https://doi.org/10.6084/m9.figshare.32685855, 1 frame every 10 s) and samples treated with 350 µM nocodazole (Noc): (**A**) nucleus blocked at a septum (Movie 13 at https://doi.org/10.6084/m9.figshare.32685855, 1 frame every 27 s); (**B**) nucleus blocked between the spermatium and the trichogyne (Movie 14 at https://doi.org/10.6084/m9.figshare.32685855, 1 frame every 24 s); (**C**) nucleus floating freely in the cytoplasm (Movie 15 at https://doi.org/10.6084/m9.figshare.32685855, 1 frame every 30 s). In the control panel, both spermatia with their H1-mCherry-labeled nuclei are visible. In panel Noc-b, an asterisk indicates an H1-mCherry-labeled nucleus remaining inside the spermatium. In panel Noc-c, a spermatium is observed contacting the trichogyne tip with its H1-mCherry-labeled nucleus still inside. MT depolymerization by nocodazole prevents male nucleus migration and abolishes nuclear stretching. TL: transmitted light. Time: min:s. Scale bar: 10 µm. (**B**) Front speed of male nuclei. Speed was measured at each time point for all nuclei tracked in the time-lapse recordings. Mean speeds are indicated in the graph. Nocodazole treatment causes a dramatic collapse in male nuclei speed. *N*_*N*_: number of nuclei analyzed; *n*: number of front speed measurements. (**C**) Maximum stretching of male nuclei. For each nucleus analyzed, the longest stretching observed during the recording was measured. Mean stretching lengths are shown in the graph. No nuclear stretching is observed under nocodazole treatment. *N*_*N*_: number of nuclei analyzed. Control was treated with 1.1% DMSO. Welch’s *t*-test *P*-values: ns = not significant; *P < 0.05; **P < 0.01; ***P < 0.001; ****P < 0.0001.

As shown above, actin depolymerization did not affect male nucleus movements. To investigate whether MTs are involved in these movements, we crossed *H1-mCherry* as male with β*-Tubulin-GFP* as female and analyzed the behavior of migrating male nuclei in relation to the MT network within trichogynes. We found that when H1-mCherry-tagged male nuclei were in a relaxed state (i.e., between stretching phases), there was no consistent or clear co-localization with GFP-tagged MTs (Fig. 8C; Movies 5 and 12 at https://doi.org/10.6084/m9.figshare.32685855). In sharp contrast, during stretching phases, we observed a clear co-localization between the front part and the stretching regions of male nuclei and MT bundles (Fig. 8C; Movies 5 and 12), suggesting that the leading edge of male nuclei was pulled along MT tracks. To functionally test the role of MTs in male nucleus migration, we depolymerized MTs using nocodazole (Noc), following the same protocol used for Lat B treatment: samples were mounted in Noc 4 h after inoculating β*-Tubulin-GFP* female partners with *H1-mCherry* spermatia, and we then monitored both male nucleus movement and the MT network. For Noc treatment (see Materials and Methods), a dilution series ranging from 32 µM to 350 µM was used to determine the optimal treatment conditions (Fig. S4). As shown in Fig. S4, microtubule bundles remained detectable up to 175 µM Noc. Therefore, subsequent Noc treatments were performed at 350 µM, a concentration that induced immediate microtubule depolymerization. Noc treatment led to a rapid and nearly complete depolymerization of GFP-labeled MT bundles (Fig. 10A; Movies 13 to 15 at https://doi.org/10.6084/m9.figshare.32685855). Strikingly, while 13 out of 13 nuclei migrated in untreated control β*-Tubulin-GFP* trichogynes, none of the 11 observed nuclei migrated in Noc-treated trichogynes. Moreover, these nuclei exhibited no stretching phases and maintained a constant length of 5.9 ± 1.3 µm similar to the size of the relaxed state of male nuclei (Table S1; Fig. 10C). Notably, in the absence of MTs, male nuclei were not spherical but retained an irregular, loose shape. They were often found partially blocked or compressed within septal pores (Fig. 10Aa; Movie 13) or arrested at the entry point of the trichogyne (Fig. 10Ab; Movie 14). When free in the cytoplasm (Fig. 10Ac; Movie 15), male nuclei exhibited only residual, uncoordinated movements, in stark contrast to the active migration observed in untreated samples (Fig. 10A, control; Movies 5, 10, 12 and S2 at https://doi.org/10.6084/m9.figshare.32685855). Together, these results demonstrate that MT depolymerization by Noc severely impairs male nucleus movement and stretching, indicating that both migration and deformation of male nuclei within trichogynes are MT-dependent processes.

## DISCUSSION

Nuclear dynamics and deformation have been studied in various model systems, including yeast, *Drosophila*, and human cells, using micropatterning approaches (12, 14, 35). In this study, we explored the behavior of both female and male nuclei during sexual reproduction in the model filamentous fungus *P. anserina*, with a particular focus on male nucleus migration. The most striking observation was the remarkable ability of male nuclei to move, stretch, and contort as they migrate through the female trichogyne, mimicking inchworm movement. The conservation of this phenomenon in both *P. anserina* and *N. crassa* highlights the potential of filamentous fungi as robust models for studying extreme nuclear dynamics and deformation *in vivo*. Male nucleus migration is characterized by (i) exceptionally high migration speed, (ii) dramatic stretching, particularly when navigating through obstacles such as female nuclei and septal pores, (iii) the ability to perform back-and-forth movements, and (iv) the capacity to select the correct path toward the protoperithecium. Furthermore, we observed that female nuclei dynamics within trichogynes is significantly reduced during male nucleus migration. This mirrors findings in *N. crassa* and suggests a conserved inhibitory effect of male nucleus migration on female nucleus motility (17). Further studies focusing on female nuclei will be required to elucidate what controls female nuclei oscillatory movements and how they are affected upon male nucleus migration.

We frequently observed that multiple male nuclei can migrate simultaneously within trichogynes and eventually enter into a single protoperithecium, leading to what we called polyfertilization of this protoperithecium. A detailed analysis of this protoperithecial fertilization revealed, indeed, that 50% of fertilized protoperithecia received more than one male nucleus. Upon entry into the protoperithecium, male nuclei ultimately accumulate in its central region—presumably the ascogonium—where they are expected to engage in the next stages of sexual development, including proliferation and meiosis, ultimately leading to ascospore production (21). In scenarios involving genetically distinct male nuclei such as in nature, one would anticipate the formation of genetically mosaic perithecia producing asci of different paternal origins. However, when females were fertilized with spermatia of different genotypes, we did not observe any mosaic perithecium. We propose two hypotheses to explain this apparent discrepancy between cytological observations and genetic outcomes. First, “polyfertilized” protoperithecia may degenerate during development and fail to mature into perithecia capable of producing ascospores. Second, the ascogonium has not yet been specifically identified due to the lack of molecular markers, and thus, we cannot definitively determine whether all male nuclei entering the protoperithecium also enter the ascogonium. It remains possible that only one male nucleus successfully enters the ascogonium, while supernumerary nuclei are excluded before the onset of the subsequent developmental stages. In support of this, it was recently shown that once fertilization has occurred, perithecia in *P. anserina* become refractory to further fertilization within a few hours (36). Further studies will be necessary to elucidate the fate of male nuclei within the protoperithecium. Answering these questions will require significant advances in live imaging of nuclear dynamics within the protoperithecial core. Beyond nuclear proliferation, several key processes take place at this stage, including internuclear recognition prior to karyogamy and meiosis (22), as well as genome defense mechanisms such as Repeat-Induced Point mutation (RIP) which remains poorly characterized in *P. anserina* (37). High-resolution, live-cell imaging of these events will be crucial to uncover the spatial and temporal dynamics governing nuclear behavior and genome regulation during sexual development in filamentous fungi.

In this study, we investigated whether the actin or microtubule (MT) cytoskeletal networks are responsible for enabling the striking movements and deformations of male nuclei during their migration through trichogynes. Although both cytoskeletal components are more prominent in trichogynes than in vegetative hyphae, our data strongly support a predominant role for MTs in this process. While actin cables were occasionally co-localized with stretched male nuclei, the depolymerization of actin filaments using latrunculin B did not impair male nucleus migration or stretching, arguing that the actin cytoskeleton does not play a major role in these dynamics. In contrast, MTs form extensive and dynamic bundles throughout the trichogyne. We show that in trichogynes (after their growth phase), most of the MTs likely originate from the septal MTOC, but we also show that MTs cross septal pores as observed in other species (38, 39). Moreover, we show that their depolymerization using nocodazole results in a complete arrest of male nucleus migration and a loss of stretching behavior. This clearly demonstrates that both movement and deformation of male nuclei in trichogynes are MT-dependent. The fact that the front side of stretched male nuclei colocalize with MTs bundles suggests that male nuclei are pulled along MT tracks that may extend across septal pores, providing a structural and functional basis for their directed migration.

**Fig 11:**
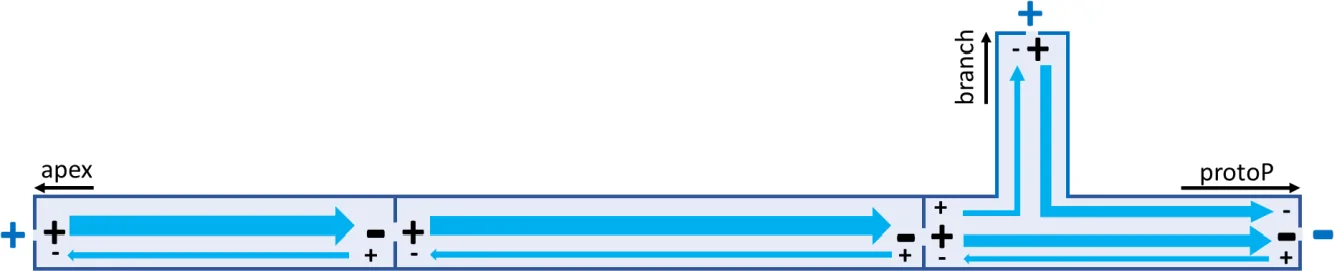
Microtubule minus-end-directed nuclear movements in trichogynes. In trichogynes, MTs are predominantly nucleated at septal microtubule-organizing centers (MTOCs) in a biased manner, with most elongating toward the apices of the branches. This generates a polarized MT network at both the cellular compartment level and along the entire trichogyne, with the majority of MT plus-ends oriented toward the apex and minus-ends toward the protoperithecium (protoP). Although male nuclei may also be capable of migrating toward MT plus-ends, minus-end-directed movements (blue arrows) on their own can explain how: (i) male nuclei ultimately migrate toward the protoperithecium following the overall polarity of the MT network (thick arrows); (ii) how they undergo back-and-forth movements by switching between MTs of opposite orientation; and (iii) how they can navigate branching points effectively, as nuclei that enter an incorrect branch (thin arrows) eventually reverse direction and reorient toward the protoperithecium along the dominant MT polarity (thick arrows).

One of the most intriguing abilities of male nuclei is their capacity to orient themselves within branched trichogynes, effectively choosing the “correct path” toward the protoperithecium and reversing direction when initially taking a wrong turn at branch points. Our study provides important insights into how male nucleus may achieve this orientation by analyzing MT dynamics. We first observed a striking bias in the direction of MT bundle growth, with the majority extending toward the apex extremity of the trichogyne. This suggests a polarized MT network in which most MT plus-ends are oriented in the direction of the apex, whereas MT minus-ends are located on the proximal side of each trichogyne article (cell). In fungal cells, the main microtubule organizing centers (MTOCs) are the spindle pole bodies (SPBs) associated with nuclei and septa (32–34). We focused on septa due to their pronounced β-tubulin-GFP tagging observed in trichogynes. Unlike in vegetative hyphae, where MT growth at septa is infrequent in *P. anserina*, we detected numerous MT bundle extensions at septa in trichogynes. Since specific tagging of MT plus or minus-ends is unavailable in *P. anserina*, we counted the MT bundles growing in the direction of the apex or in the protoperithecium direction at septa, in native conditions and in FRAP experiments. Determining the directionality of these MT bundle extensions allowed us to infer the overall MT polarity within trichogyne compartments. This polarized MT network is consistent both before plasmogamy and during male nucleus migration. These observations strongly suggest that male nucleus migration is predominantly a minus-end-directed movement along MTs toward the protoperithecium. Without to exclude the possibility that nuclei may also have an anterograde movement (toward the plus-ends of MTs; see below), we propose a model in which male nuclei can navigate branched trichogynes by following the predominant MT minus-end polarity, which effectively guides them retrogradely to the protoperithecium (Fig. 11). Occasionally, the minority of MTs with minus-ends oriented toward branch apices could misdirect nuclei, causing them to initially migrate in the wrong direction. In this model, the ability of male nuclei to change direction at branch points or within compartments depends on two key properties: (i) performing retrograde (minus-end-directed) movements and (ii) switching between MT bundles oriented in opposite directions. Ultimately, the global polarization of MTs, with minus-ends pointing toward the protoperithecium, ensures that nucleus migration is biased in the correct direction.

Regarding the molecular machinery driving this movement, although microtubule-associated molecular motors have not been studied in *P. anserina*, genomic analysis has identified putative kinesins and components of the dynein/dynactin complex (40). While kinesins capable of retrograde transport cannot be excluded, the dynein/dynactin complex—known universally to mediate retrograde transport along MTs in eukaryotes (13, 32, 41, 42)—represents a likely candidate driving male nucleus migration toward the protoperithecium. Even though our model based on the minus-end directed movement of male nuclei can recapitulate the tortuous male nucleus movements in trichogynes (Fig. 11), we do not exclude that anterograde movements (toward MT plus-ends) involving kinesins may also contribute to male nuclei direction changes during their migration. We anticipate that functional studies of both types of MT-associated molecular motors in *P. anserina* will provide further insight into the mechanisms underlying male nucleus migration within trichogynes.

A remarkable feature of migrating male nuclei in fungi is their exceptionally high migration speed. Because male nuclei in trichogynes are not spherical, we defined distinct regions—front, center, and rear—to accurately describe their dynamics and quantify velocity. Since nuclear movements are presumably driven by MT-associated molecular motors, the speed of the nuclear front, which corresponds to active traction, serves as the most relevant parameter to estimate the motor velocity. The highest front speed measured in our study was 2.1 µm/s (125 µm/min), a value comparable to dynein-driven retrograde transport speeds reported in *Ustilago maydis* (43), yet strikingly fast for nuclear migration in general (44).

We also demonstrate that male nuclei change direction within trichogynes through two distinct mechanisms: by performing U-turns or by reversing their movement (i.e., backward migration). Assuming that male nucleus movements result from pulling forces generated by molecular motors anchored to the nuclear envelope (NE) at the leading edge of the nucleus, these two modes of directional change raise important questions about the spatial distribution of such pulling forces. In the case of U-turns, the front of the nucleus changes direction, implying that a single pulling site localized at the nucleus front drives this reorientation. Conversely, reversing movement suggests that an additional pulling site located on the opposite, rear side of the nucleus becomes engaged to mediate backward migration. In metazoans, the LINC complex mechanically couples the NE to cytoskeletal motors, thereby transmitting forces necessary for nuclear positioning and movement. The LINC complex has been studied in both yeasts *Saccharomyces cerevisiae* and *Schizosaccharomyces pombe* where it is involved in SPB functioning (i.e., duplication and insertion into the NE), NE homeostasis, chromatin tethering to the NE, and chromosomes movements during meiosis (45–56). Whether the LINC mediates pulling forces driving male nucleus movements in trichogynes remains an important open question.

A further remarkable characteristic of male nucleus migration is the astonishing elongation phases, which reflect the exceptional flexibility of male nuclei. In their relaxed state, male nuclei exhibit a loose, irregular shape that contrasts markedly with the more compact morphology of female nuclei within trichogynes or of vegetative nuclei. Upon stretching, their length can increase dramatically (from approximately 6 µm to as much as 71 µm), representing nearly a 12-fold elongation. This flexibility is particularly crucial when traversing septa: male nuclei must squeeze through pores estimated to be around 200 nm in diameter despite having a width of roughly 800 nm in their relaxed form, about four times larger than the pore size. We show that these elongation phases depend on MTs, suggesting that the stretching results from pulling forces exerted by MT-associated motor proteins. Supporting this hypothesis, we observed clear co-localization of the leading edge of male nuclei with MT bundles during stretching phases. Conversely, we hypothesize that uncoupling of the nucleus from MTs leads to nuclear relaxation and cessation of migration. Consistent with this, no co-localization with MT bundles at the extremities of male nuclei was detected during periods when male nuclei exhibited a relaxed morphology.

The migration of male nuclei is a long-range process during which they repeatedly squeeze through septal pores and undergo significant stretching, thereby facing intense and repetitive mechanical stress. Remarkably, we have never observed any male nucleus rupturing during migration, indicating that this process is highly robust and suggesting that male nuclei are structurally adapted to withstand such mechanical challenges. One of the clearest adaptations is their loose and irregular shape. Importantly, this specific shape appears independent of the actin and MT cytoskeletons, as male nuclei retain their characteristic morphology even when both cytoskeletal networks are depolymerized. This suggests that this unique nuclear flexibility is an inherent feature of the structure and composition of the male nucleus itself. In metazoans, lamins play a central role in determining nuclear stiffness and shape; however, lamins are absent in fungi, and to our knowledge, no other nuclear components have been demonstrated to fulfill a similar structural role in fungal nuclei (7, 57). While the lack of lamins might partially explain the looseness of fungal nuclei in general, it does not clarify why this loose shape is specific to male nuclei and not observed in vegetative or female nuclei. Various factors may contribute to the enhanced flexibility of male nuclei, including differences in NE composition (both lipids and proteins) as well as chromatin organization and its potential tethering to the NE (5, 58). Furthermore, the extremely short time interval between plasmogamy and the onset of male nuclei entry into the trichogyne suggests that male nuclei are primed for migration at the time of plasmogamy. Consequently, the characteristic features of male nuclei may be established before plasmogamy occurs. Future work aimed at identifying the molecular determinants of male nuclei flexibility and robustness will provide valuable insights into nuclear mechanotransduction and adaptation to mechanical stress.

Movements and deformations of the fungal nucleus during sexual reproduction are remarkably complex, representing some of the most extreme nuclear dynamics described across all eukaryotes. A lot of work remains to be done before to fully characterize these processes. We propose that studying mechanotransduction in male nuclei during sexual reproduction in the fungus *Podospora anserina* offers a powerful model to investigate the molecular mechanisms enabling their exceptionally rapid movement. This research could reveal how nuclei endure extreme mechanical stresses and uncover the potential impacts of these forces on chromatin organization and gene expression.

## MATERIALS AND METHODS

### Strains and culture conditions

The strains used in this study are all listed in Table 1. All *P. anserina* strains derive from the wild-type *S* strain, ensuring a (Table 1) homogeneous genetic background (40, 59). The *PaPks1^193^* mutant, deficient for the polyketide synthase-encoding gene acting at the first step of melanin synthesis, is described in reference 24. Standard culture conditions, media, and genetic methods for *P. anserina* were described in references 18, 23 and on the *Podospora anserina* database (http://podospora.i2bc.paris-saclay.fr/). The M0 medium is similar to the M2 medium but without dextrin.

### Strains construction

#### H1-GFP, H1-mCherry, and H1-mCherry GFP^cyto^

The genomic sequence of the putative H1 histone (*Pa_5_8740*), including its 854 bp upstream regulatory region, was amplified using the Phusion polymerase (https://www.thermofisher.com) with primers H1-TAGF (5′-CTCGAGCAGCTTAGCGGCAGATCGAG-3′), bearing an *X*hoI site at the 5′ end, and H1-TAGR (5′-AAGCTTCGCCGAGGCAGCTTCCGCCT-3′), bearing a *H*indIII site at the 5′ end, and omitting the stop codon of *Pa_5_8740* CDS. 3′-OH thymidines were added in a subsequent step with the GoTaq polymerase to enable cloning into pGEM-T (https://france.promega.com/). The PCR product was cloned into pGEM-T, digested with *X*hoI and *H*indIII (FastDigest, https://www.thermofisher.com), and inserted into pBC-hygR-eGFP or pBC-hygR-mCherry digested with the same enzymes, creating the pBC-hygR-H1-GFP and pBC-hygR-H1-mCherry transformation vectors conferring hygromycin B resistance [hygR]. Both vectors were transformed into the wild-type *S* strain, and two independent [hygR] primary transformants per construct were selected based on nuclear fluorescence intensity. Subsequently, the respective *H1-GFP* and *H1-mCherry* primary transformants (named so for simplicity) were backcrossed with the *S* strain, and homokaryotic progeny of each mating type (*mat*+ and *mat*−) were selected. Only one *H1-mCherry* and one *H1-GFP* transformant were used in this study. Double-tagged *H1-mCherry GFP^cyto^* strain was obtained by crossing *H1-mCherry* strain with *GFP^cyto^* strain (25) and selecting homokaryotic [hygR]—both strains carry the hygromycin B resistant gene—progeny of each mating type showing the double tagging of the cytoplasm (in green) and of the nucleus (in red).

#### β*-Tubulin-GFP* and β*-Tubulin-mCherry*

Both constructs, composed as follows—<*S*peI site–444 bp regulatory sequence of the *AS4* gene (strong, constitutive *AS4* promoter [25, 60])–GCCACC (Kozak sequence)*–Pa_4_7630* CDS (putative β-tubulin) without stop codon*–eGFP* or *mCherry* CDS*–S*peI site>—were synthesized by IDT (https://eu.idtdna.com/). Both pUCIDT-β-Tubulin-GFP and pUCIDT-β-Tubulin-mCherry were digested by *S*peI (FastDigest, https://www.thermofisher.com), and the inserts were subcloned into pBC-phleoR (conferring phleomycin resistance) digested with *S*peI, generating the pBC-phleoR-β-Tubulin-GFP and pBC-phleoR-β-Tubulin-mCherry transformation vectors. These vectors were transformed into the wild-type *S* strain, and two independent [phleoR] primary transformants per construct were selected based on MT network fluorescence intensity. The primary transformants were backcrossed with the *S* strain, and homokaryotic progeny of each mating type (*mat*+ and *mat*−) were selected. These strains were named β*-Tubulin-GFP* and β*-Tubulin-mCherry* for simplicity. Only one β*-Tubulin-GFP* transformant was used in this study. β*-Tubulin-GFP* was crossed with *H1-mCherry*, and [hygR phleoR] *H1-mCherry* β*-Tubulin-GFP* double-tagged homokaryotic progeny of each mating type were selected.

#### LifeAct-GFP

Two long reverse complementary oligonucleotides of the following sequence: AAGCTTGCCACCATGGGCGTCGCCGACCTCATCAAGAAGTTCGAGTCCATCTCCAAGGAGGAGGGGGCCC, composed as follows—<*Hi*ndIII site–GCCACC (Kozak sequence)*–Neurospora crassa* Lifeact sequence (29) encoding the Lifeact 17 AA actin-binding peptide (MGVADLIKKFESISKEE) (27) with the codon GCT>A changed to GCC>A (underlined)–additional G (underlined) for in-frame cloning*–A*paI site>—were synthesized by IDT (https://eu.idtdna.com/). A mix of both oligonucleotides was used for direct 3′-OH thymidine addition by GoTaq (https://france.promega.com/) followed by cloning into pGEM-T (https://france.promega.com/). pGEM-T-LifeAct-GFP was digested by *H*indIII and *A*paI (FastDigest, https://www.thermofisher.com), and the insert was subcloned into the pBC-hygR-AS4-GFP transformation vector digested by *H*indIII and *A*paI (25). In the pBC-hygR-AS4-LifeAct-GFP vector, transcription of the LifeAct-GFP reporter is driven by the strong constitutive *AS4* promoter. This vector was transformed into the wild-type *S* strain, and one [hygR] primary transformant was selected based on actin network fluorescence intensity. The primary transformant was backcrossed with the *S* strain, and homokaryotic progeny of each mating type (*mat*+ and *mat*−) were selected. The strain was named *LifeAct-GFP* for simplicity. *LifeAct-GFP* was crossed with *H1-mCherry*, and [hygR] homokaryotic *H1-mCherry LifeAct-GFP* progeny of each mating type, exhibiting both nuclei (red) and actin (green) tagging, were selected.

#### PH-mCherry

The AS4-PH-mCherry construct, composed as follows—<*S*peI site–444 bp *AS4* promoter–GCCACC (Kozak sequence)–552 bp *Homo sapiens* Pleckstrin-homology (PH) plasma membrane phosphoinositide-binding domain (61)–mCherry CDS–*S*peI site>—was synthesized and cloned into pUCIDT(Amp) by IDT (https://eu.idtdna.com/). The pUCIDT-AS4-PH-mCherry vector was digested with *S*peI (FastDigest, https://www.thermofisher.com), and the insert was subcloned into the pBC-nouR transformation vector (conferring nourseothricin resistance) digested with *S*peI. pBC-nouR-AS4-PH-mCherry was transformed into the wild-type *S* strain, and two [nouR] primary transformants were selected based on plasma membrane fluorescence intensity. These transformants were backcrossed with the *S* strain, and homokaryotic progeny of each mating type (*mat*+ and *mat*−) were selected. The strain was named *PH-mCherry* for simplicity. Only one *PH-mCherry* strain was used in this study. This strain was crossed with *H1-GFP*, and homokaryotic [hygR nouR] *H1-GFP PH-mCherry* progeny of each mating type were selected.

### Live-cell imaging

All the movies are downloadable from Figshare: https://figshare.com/; DOI:10.6084/m9.figshare.32685855

### Drug treatment setup

The working concentrations of latrunculin B (Lat B) and nocodazole (Noc) were first established using vegetative hyphae expressing the LifeAct-GFP reporter for Lat B treatment and the β-tubulin-GFP reporter for Noc treatment. The standard concentration for fungi of 25 µM Lat B (stock solution: 8 mM in DMSO) (30) induced an almost immediate and complete depolymerization of actin in both vegetative hyphae and trichogynes (data not shown for vegetative hyphae; Figure 7 and Movie 9 at https://doi.org/10.6084/m9.figshare.32685855 for trichogynes). For Noc treatment, a dilution series ranging from 32 µM to 350 µM (stock solution: 30 mM in DMSO) was used to determine the optimal treatment conditions (Fig. S4). Observations of vegetative hyphae and trichogynes were performed on inverted agar blocks. Depending on the depth of the observed hypha or trichogyne within the agar, drug penetration and treatment efficiency may vary. Consequently, during fertilization experiments, only trichogynes displaying clear actin or microtubule depolymerization, as evidenced by the loss of the LifeAct-GFP or β-tubulin-GFP fluorescence pattern, were considered for analysis.

### Vegetative hypha live imaging

The strains analyzed were grown on solid M2 medium, and samples were mounted inverted in water in 2-well µ-Slide ibidiTreat chambers (https://ibidi.com/). Images were acquired using a motorized inverted microscope Zeiss spinning disk CSU-X1, as described below.

### Fertilization procedure

Petri dishes containing solid M0 medium were inoculated with the “female” strain (*mat*− or *mat+*) and incubated for 5–7 days to allow protoperithecia development. The inoculum was placed on a 3 × 3 cm piece of 3M paper (https://www.3mfrance.fr) serving as a carbon source, and observations were performed on the mycelium growing in the translucent M0 medium. Under these conditions, mycelium density is reduced, which facilitates microscopic observation while allowing sexual development to proceed. A 2–3 cm² piece of M0 medium containing protoperithecia was cut and transferred to an empty petri dish. This sample was inoculated with 100–200 µL of a 1–2 × 10⁶ spermatia/mL suspension of the “male” partner of the opposite mating type, in sterile H₂O with 0.01% Tween 20. Spermatium suspensions were prepared on the day of inoculation.

### Fertilization live imaging

After 4 h of incubation at 27 °C in a moist chamber, the sample was mounted inverted in one of the following conditions: sterile water, 350 µM nocodazole, 1.1% DMSO (nocodazole control), 25 µM latrunculin B, or 0.3% DMSO (latrunculin B control), using a 2-well µ-Slide ibidiTreat chamber. Samples were then inspected for fertilization events. Images were acquired using a Zeiss spinning disk CSU-X1 motorized inverted microscope (Oberkochen, Germany), equipped with four lasers (405 nm, 488 nm, 561 nm, 640 nm) for fluorescence imaging and their respective filters, and a sCMOS PRIME95 camera (Photometrics), at the ImagoSeine Imaging Facility: https://www.ijm.fr/plateformes-et-plateaux-techniques/imagoseine/. For low-magnification imaging (×25 and ×40 objectives), z-stacks covering a 50 µm range were acquired and processed into z-projection images. Time-lapse acquisition was carried out over 5–10 h with a frame rate of 1 image every 3–5 min. For high-magnification imaging (×63 objective), time-lapse acquisition was performed over 5–20 min with a frame rate of 1 image every 6–30 s and a variable z-range of 6–25 µm. Z-stacks were processed into z-projection images, except for co-localization analyses. Images were analyzed with FIJI (62), except for quantification of female nucleus movements, which was performed using IMARIS (https://imaris.oxinst.com/).

### Male nucleus speed measurements

The speed of the front, center, and rear of male nuclei was measured manually at each time point of the time-lapse recordings. Front speeds were most frequently analyzed in this study. The highest front traction speed determined was 125 µm/min based on image-by-image analysis. For each time point, we verified whether the measured front speed (only values above 100 µm/min were considered significant) corresponded to a traction or retraction movement. Rear speeds were analyzed similarly, and the highest rear speed recorded was 248 µm/min, corresponding to a nuclear retraction. For nocodazole-treated nuclei that were blocked during migration without stretching, front speed was measured by selecting one extremity of the nucleus. For male and female nuclei speed analyses, the number of nuclei analyzed (*N*_*N*_) and the number of individual speed measurements (*n*) are indicated in the figures. For statistical analysis, speed values measured from different nuclei under the same condition were pooled. Conditions were compared using Welch’s *t*-test. *P*-values are indicated as follows: ns, not significant; *P < 0.05; **P < 0.01; ***P < 0.001; ****P < 0.0001.

### MT elongation quantification

MT polarization in vegetative hyphae and trichogynes, both before and after plasmogamy, was determined by counting visible MT elongations at septa. The speed of the MT bundles growth was measured manually at each time point of the time-lapse recordings and presented in Fig. 9F. For quantification, we analyzed the most β-tubulin-GFP-tagged focal plane (corresponding to the most central z-section) and recorded MT elongations live over a 10-min period. In total, 23 septa were analyzed in vegetative hyphae, 13 in trichogynes before plasmogamy, and 8 in trichogynes after plasmogamy during male nucleus migration. For each septum, the number of MT elongations was counted, and the mean number is presented in Fig. 9B. MT elongations directed toward the apex and those directed toward the protoperithecium were counted separately. The percentage of elongations oriented toward the apex in trichogynes is also presented in Fig. 9C.

### FRAP

FRAP experiments were performed at septa in trichogynes prior to male nucleus migration. Imaging was conducted on a single focal plane, at five septa located in five different trichogynes. The selected focal plane corresponded to the most central plane displaying the highest β-tubulin-GFP fluorescence signal. Before photobleaching, a 30 s time-lapse recording was acquired at a frame rate of one image every 5 s. Photobleaching was then performed by illuminating the region of interest (11–18 µm in length) four times for 40.8 ms each. Following photobleaching, images were acquired for 4 min at a frame rate of one image every 4 s. Images were obtained using a Zeiss spinning-disk CSU-X1 motorized inverted microscope (Oberkochen, Germany) equipped with four lasers (405, 488, 561, and 640 nm) for fluorescence imaging, their corresponding filter sets, and a PRIME95 sCMOS camera (Photometrics). Image analysis was performed using FIJI. Trichogynes were oriented with the apex positioned to the left (Fig. 9D; Movie S7 at https://doi.org/10.6084/m9.figshare.32685855). During the 4-min post-bleaching recording, the number of MT bundles growing in the apical direction (leftward) or in the direction of the protoperithecium (rightward) was manually quantified (Fig. 9E). In some of the movies, MTs emerging within the photobleached region could be identified most reliably on the proximal side of the septum (right side). Therefore, MT quantification was systematically performed in this region for all samples. The overall percentage of MTs growing in the apical direction vs in the direction of the protoperithecium was calculated on the 30 MT bundles counted.

## SUPPLEMENTAL MATERIAL

Supplemental Material mbio.01455-26-s0001.pdf Figures S1 to S4; Table S1.

ASM does not own the copyrights to Supplemental Material that may be linked to, or accessed through, an article. The authors have granted ASM a non-exclusive, world-wide license to publish the Supplemental Material files. Please contact the corresponding author directly for reuse.
