# Supplementary material for: Fast and tortuous: extreme deformations and fast movements of the cell nucleus are driven by microtubules in the model fungus Podospora anserina
Source: mBio. 2026 Jul 31;17(9):e01455-26. doi: 10.1128/mbio.01455-26 (PMC13556228; doi:10.1128/mbio.01455-26)
Supplement: Supplementary file 1 [file mbio.01455-26-s0001.pdf]

A

fertilized perithecia			paternal contribution in perithecia			
1 nucleus	>1 nucleus	total	*wt*	*PaPks1*^{193}	mosaic	total
8	8	23	102	56	0	158
35%	35%		65%	35%	0%	

B

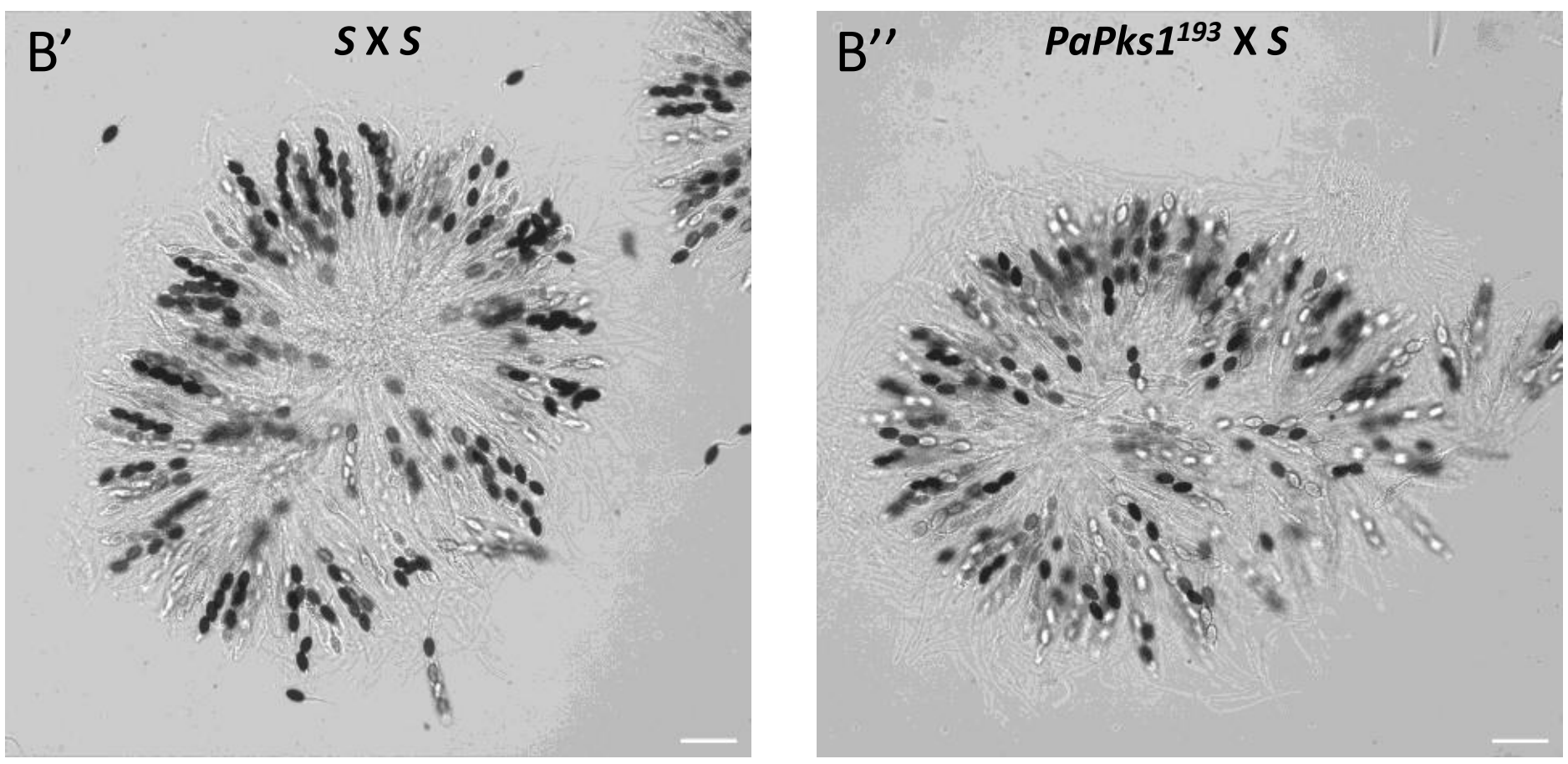

Figure S1. Polyfertilization of protoperithecia. A) Fertilized perithecia from a cross between male *H1-mCHerry* X female *wild-type*. A total of 23 protoperithecia were imaged for 15 hours (1 image every 5 minutes), and the number of male nuclei entering each protoperithecium was counted. Paternal contribution in mature perithecia was assessed by fertilizing wild-type *S* females with a mixture of wild-type *S* and *PaPks1*^{193} mutant spermatia. A total of 158 mature perithecia were dissected, and the ascus composition was determined. "wt" refers to perithecia containing only asci from *S* X *S* crosses (as shown in B'), "*PaPks1*^{193}" indicates perithecia with asci exclusively from *PaPks1*^{193}× *S* crosses (as shown in B''), and "mosaic" corresponds to perithecia containing a mix of both types of asci. **B)** Rosettes of asci from *S* X *S* crosses (B') and *PaPks1*^{193} X *S* crosses (B''). In *P. anserina*, most asci contain four heterokaryotic ascospores. In *S* X *S* crosses, rosettes consist of asci with four melanized *PaPks1*$^{+}$/*PaPks1*$^{+}$ascospores. In *PaPks1*^{193} X *S* crosses, rosettes contain asci with two melanized *PaPks1*$^{+}$/*PaPks1*$^{+}$ascospores and two non-melanized *PaPks1*^{193}/*PaPks*^{193} ascospores. Scale bar: 100 µm.

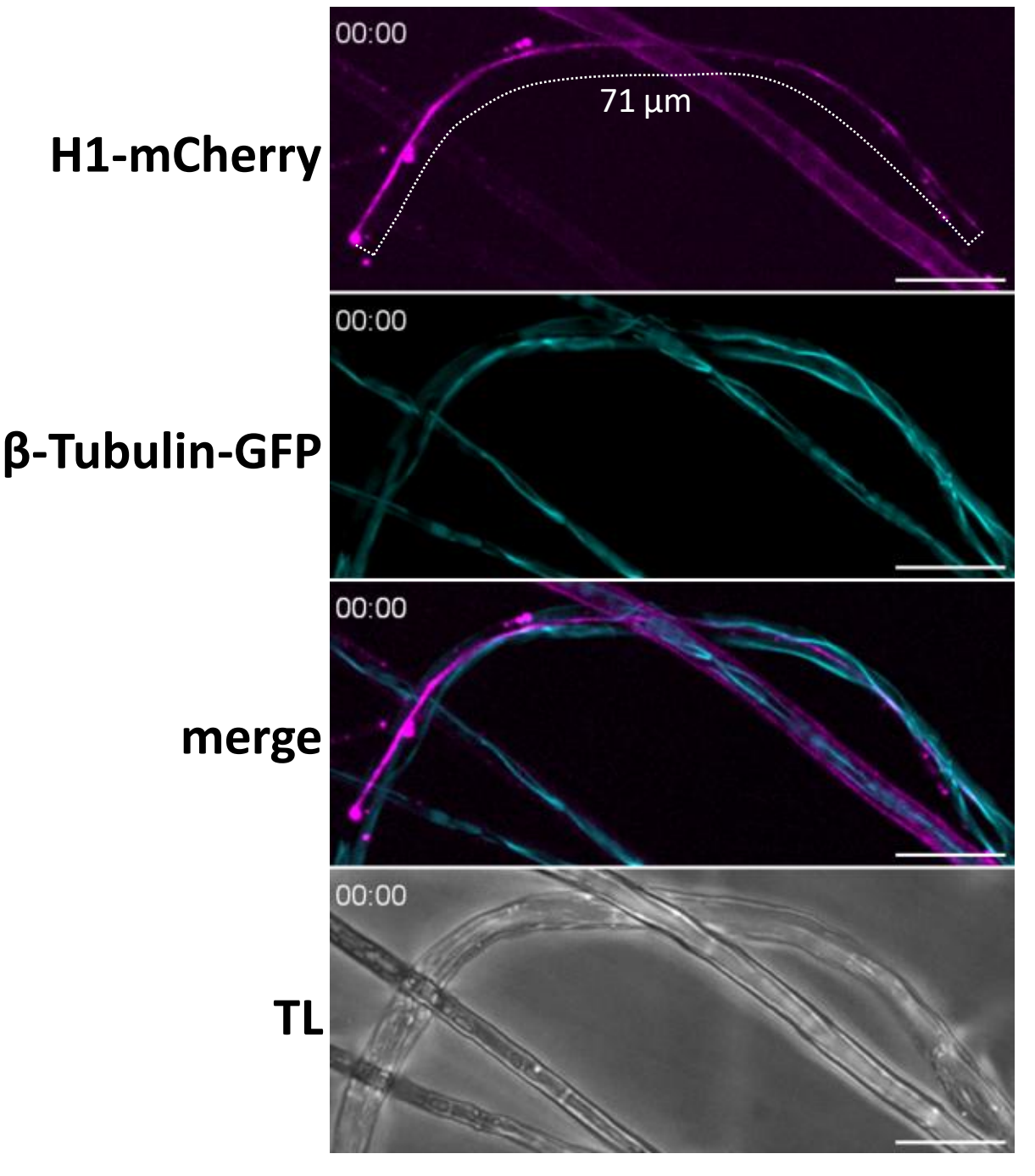

Figure S2. Longest observed stretching of a male nucleus. Cross between male *H1-mCherry* and female *β-Tubulin-GFP*. Z-projection images from time-lapse movie S2 recorded using a spinning disk microscope (frame rate: 1 image every 8 seconds). The maximum length observed for the stretched male nucleus is 71 µm. TL: transmitted light. Scale bar: 10 µm.

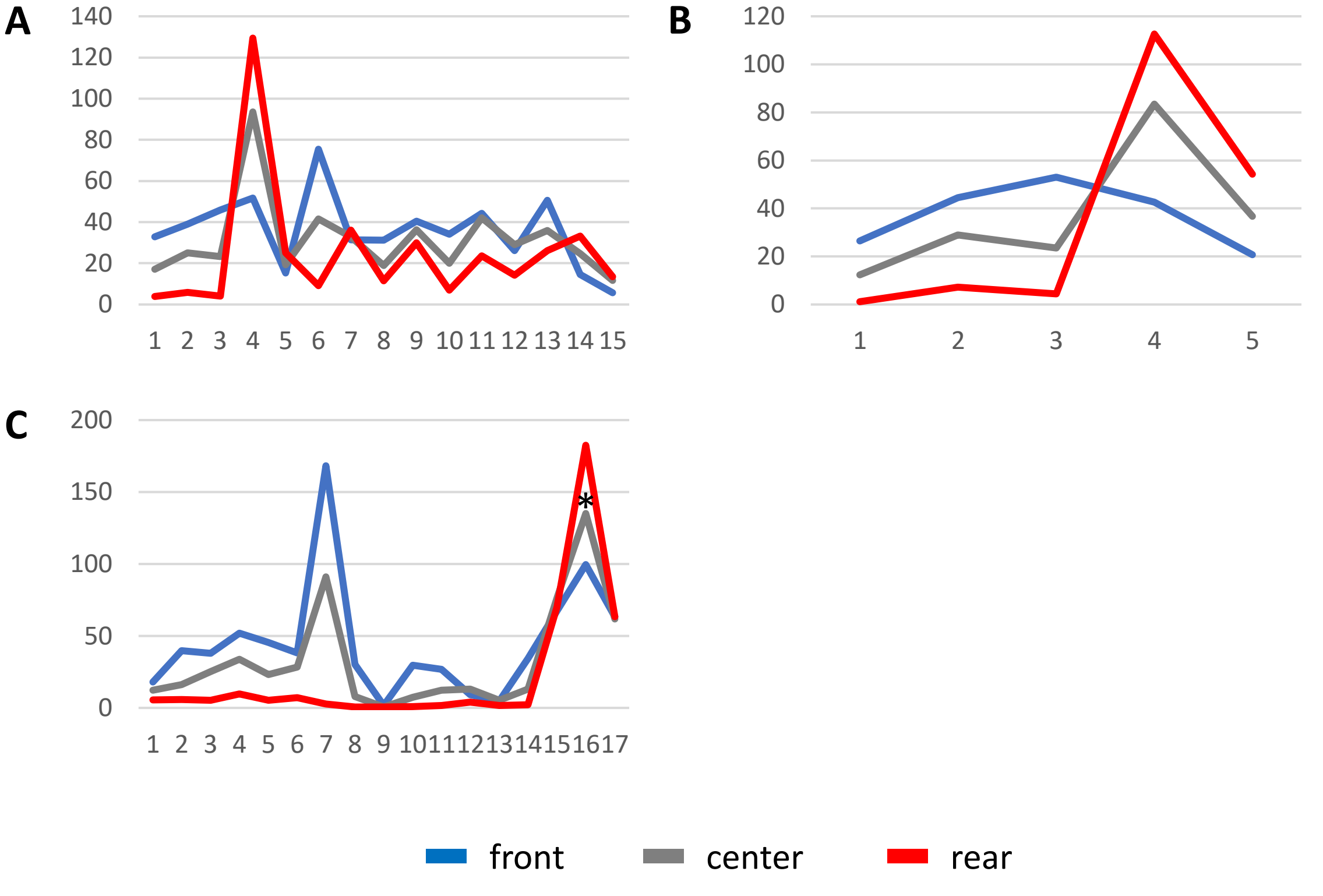

Figure S3. Comparison of front, center, and rear speeds during male nuclei stretching. Speeds are given in µm/min for each analyzed time point, restricted to intervals corresponding to nuclear stretching phases. A) male *H1-GFP* X female *PH-mCherry* (movie S3); B) male *H1-mCherry* X female *LifeAct-GFP* (movie 8); **C)** male *H1-mCherry* X female *β-Tubulin-GFP* (movie 5). The asterisk (*) highlights the highest center speed recorded in the study: 135.2 µm/min.

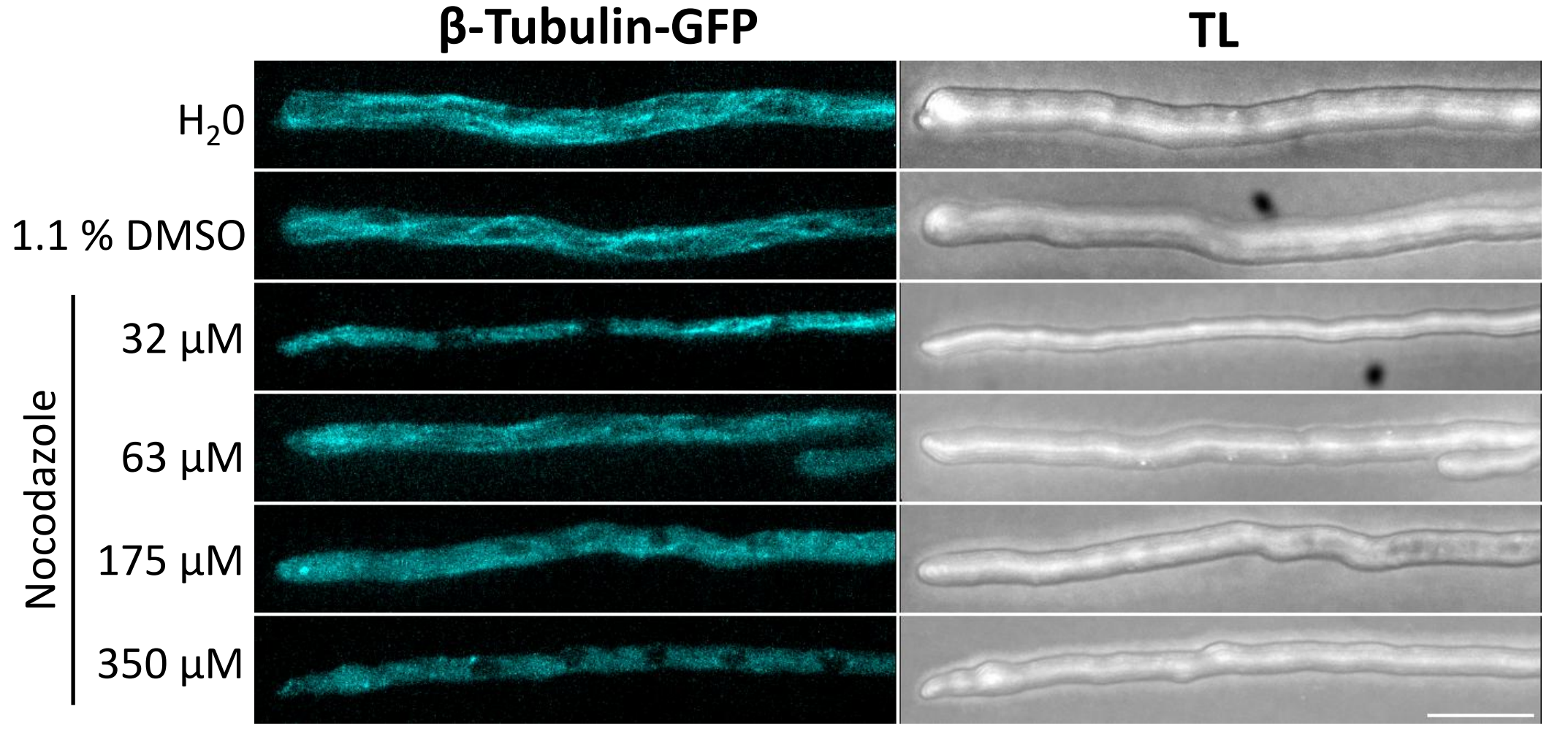

Figure S4. Nocodazole treatment set-up. Apical vegetative *β-Tubulin-GFP* hyphae grown on M2 medium. MTs bundles remain detectable up to 175 µM Noc. Therefore, subsequent Noc treatments were performed at 350 µM, a concentration that induces immediate microtubule depolymerization. The 1.1% DMSO control corresponds to the quantity present in 350 µM Nocodazole. TL, Transmitted Light. Scale bar: 10 µm.

vegetative hyphae	growth rate* (µm/min)	diameter (µm)	nuclei diameter (µm)
mean	0.2	2.4	2.3
SD	0.1	0.3	0.2
N	9	20	9
**trichogynes**	**growth rate* (µm/min)**	**diameter (µm)**	**nuclei diameter (µm)**
mean	0.6	3.5	2.1
SD	0.3	0.6	0.2
N	6	24	10
**spermatia**	**length (µm)**	**width (µm)**	**nuclei diameter (µm)**
mean	2.7	2.0	1.8
SD	0.3	0.2	0.1
N	16	16	16
**male nuclei in trichogyne**	**mean speed (µm/min)**	**length (µm)**	**width (µm)**
(relaxed state) mean	9.7	6.2	0.8
SD	15.2	1.0	0.2
N	1345 (18 nuclei)	10	17

*: growth conditions: M0 medium + paper as carbon source

Table S1: miscellaneous data. *: growth conditions: M0 medium + paper as carbon source. SD, Standard Deviation
